# Effect of non-fluoride agents on the prevention of dental caries in primary dentition: A systematic review

**DOI:** 10.1371/journal.pone.0182221

**Published:** 2017-08-07

**Authors:** Yu Wang, Jialing Li, Weibin Sun, Huang Li, Richard D. Cannon, Li Mei

**Affiliations:** 1 Department of Preventive Dentistry, Nanjing Stomatological Hospital, Medical School of Nanjing University, Nanjing, China; 2 Department of Orthodontics, Nanjing Stomatological Hospital, Medical School of Nanjing University, Nanjing, China; 3 Department of Periodontics, Nanjing Stomatological Hospital, Medical School of Nanjing University, Nanjing, China; 4 Department of Oral Sciences, Sir John Walsh Research Institute, Faculty of Dentistry, University of Otago, Dunedin, New Zealand; Virginia Commonwealth University, UNITED STATES

## Abstract

**Objective:**

To assess the effect of non-fluoride agents on the prevention of dental caries in primary dentition.

**Materials and methods:**

Medline, Web of Science, Embase, Cochrane Library, CBM and CNKI databases were searched to identify all the relevant articles published prior to 16 December 2016. Grey literature was also searched. Randomized controlled human clinical trials in which non-fluoride agents were delivered by any method were considered.

**Results:**

Of the 1,236 studies screened, 39 full articles were scrutinized and 14 selected for inclusion in the final sample. Five chemical agents, namely arginine, casein phosphopeptide-amorphous calcium phosphate (CPP-ACP), chlorhexidine, triclosan and xylitol were investigated in these included studies. The cariostatic effects of non-fluoride agents *in vivo* were evaluated in comparison with fluoride or placebos in randomized controlled trials. There is evidence that the use of certain doses of xylitol may be effective in arresting dental caries in primary dentition. However, quantitative synthesis could not be carried out because of the clinical and methodological heterogeneity of the included studies.

**Conclusions:**

A study at low risk of bias indicated that daily use of xylitol wipes is a useful adjunct for caries control in young children, however, this conclusion should be interpreted with caution as this study had a very limited sample size. Chlorhexidine and CPP-ACP may be more effective than a placebo in managing caries in primary dentition, but their effectiveness is borderline when compared with fluoride. Arginine-containing mint confection and 0.3% triclosan varnish were found to reduce caries development in primary teeth but the evidence was at high risk of bias. High quality randomized controlled trials are needed in order to make a conclusive recommendation.

## Introduction

Dental caries, a worldwide public health problem, affects a large number of people. In recent decades, although the prevalence of dental caries has dramatically decreased with the development of diagnostic methods, prevention and treatment of caries, the situation is still of concern for high-risk individuals, especially children[1].It has been reported that approximately 30% of total caries experience occurs in the primary teeth [2], and nearly half of the children in the US suffer from dental caries before entering kindergarten [3].

Caries lesions usually develop more rapidly in primary teeth than in permanent teeth due to the differences in enamel structure and dietary habits. The enamel in primary teeth is thinner, and the surface micro-hardness is relatively lower, compared with permanent teeth [4,5]. Also, primary teeth have a less well-structured crystal arrangement and comparatively less mineralization. These differences in enamel structure may lead to the caries susceptibility and faster caries progression in primary teeth. Moreover, children’s dietary habits, such as greater consumption of sugar and acidic drinks, may also contribute to the higher caries prevalence in primary teeth [5].

The development of carious lesions has been considered as a repeated dynamic process of de-/re-mineralization, which can be arrested or reversed by preventive factors in the environment or oral hygiene practice [6]. For example, chemical agents including fluoride, xylitol, chlorhexidine, and casein phosphopeptide-amorphous calcium phosphate (CPP-ACP), have demonstrated anti-caries effects in primary dentition in a large number of *in vivo* and *in vitro* studies [7–11]. The cariostatic potential of fluoride delivered in various vehicles has been demonstrated for decades, and the beneficial effects of topical fluoride agents have been examined in a series of Cochrane systematic reviews [12–14]. However, fluoride has a dose-response relationship, and improper delivery of fluoride agents may lead to adverse effects such as fluorosis [15]. Thus, numerous clinical trials have been conducted to test the ability of non-fluoride agents to enhance or supplement the remineralizing effect of fluorides, but until now their effect and safety are not clear. There are two previous systematic reviews that each assessed the efficacy of a single product for preventing dental caries in children and adults [16,17]. The authors of these reviews only found low quality evidence to suggest that xylitol may be effective for preventing caries in the permanent teeth of children. The authors of a report from the American Dental Association (ADA) on non-fluoride caries preventive agents could not make recommendations on the prevention of early childhood caries partly because insufficient studies could be found at the time this review was conducted (April 2010) [18].

The aim of this systematic review was to assess the anti-caries effect of a variety of non-fluoride agents in primary teeth, with an updated and expanded literature database search (December 2016).

## Materials and methods

This study followed the Preferred Reporting Items for Systematic Reviews and Meta-Analyses (PRISMA) statement guidelines (www.prisma-statement.org). The review was not registered before data collection.

### Search strategy and databases

A systematic search to identify all the relevant studies was conducted on the following six databases: MEDLINE (via PubMed. No restrictions were employed on language or year of publication), Web of Science, EMBASE, CENTRAL (The Cochrane Library), CBM (Chinese Biological Medical) database and CNKI (Chinese National Knowledge Infrastructure) database. A supplemental manual search was conducted by reviewing the reference lists of the related papers and review articles. The search strategy included controlled vocabulary and free terms. It was developed for MEDLINE and adapted for the other databases (S2 Table).

Grey literature was searched on Clinicaltrial.gov, OpenGrey and the World Health Organization’s International Clinical Trial Registry Platform. All searches were firstly conducted on 25 December 2015 and updated on 16 December 2016.

### Selection criteria

Human randomized controlled clinical trials were included. Studies in which participants had carious lesions in the primary dentition or mixed dentition (outcome reported on primary teeth) at the start of the study were considered for inclusion in this review, irrespective of the baseline caries experience. The inclusion age range of participants was 0–12 years old. All carious lesions (including ICDAS 1 and 2) were included. Studies in which participants had systemic disease were excluded.

### Type of intervention reviewed

Studies using non-fluoride agents, such as arginine, chlorhexidine (CHX), xylitol, casein phosphopeptide–amorphous calcium phosphate (CPP-ACP) and bioactive glass in any modality that were compared with placebos and/or fluoride were included. No restrictions were implemented regarding the dose, frequency, duration or method of non-fluoride agent administration.

The primary outcome of studies was caries increment in primary teeth or change in the proportion of participants developing new caries on primary teeth. The secondary outcome was the side effects from using the non-fluoride agents, such as gastrointestinal complaints, pain and discomfort, tooth staining, oral hygiene deterioration, quality of life and patient satisfaction.

### Data extraction

Two calibrated reviewers (Y.W. and J.L.) screened the titles and abstracts of the identified studies independently and in duplicate. Consensus was obtained by discussion and consultation with the third reviewer (L.M.) to resolve any disagreements during study selection and data extraction. Studies not meeting the inclusion criteria were excluded, and the reasons for exclusion are noted in S3 Table. The two reviewers (Y.W. and J.L.) independently extracted data from the studies using a data extraction form. The following data were collected: author and year of publication, number and age of participants, details of interventions and controls, assessment methods and time points, primary and secondary outcomes, and follow-up period.

### Methodological quality appraisal

Each study was assessed using the evaluation method recommended by the Cochrane Handbook for Systematic Reviews for Interventions 5.1.0 (http://handbook.cochrane.org). Two reviewers (Y.W. and J.L.) appraised the studies independently according to the following aspects: random sequence generation, allocation concealment, blinding, completeness of outcome data, selective outcome reporting, and other biases. Each aspect was classified as having either a low, high, or unclear risk of bias. Thus, the overall level of risk for each study was subsequently classified as low (all quality items were met), unclear (unclear risk of bias for one or more domain), or high (high risk of bias for one or more domain).

Continuous outcomes were analysed using mean differences (MD) and standard deviations (SD), and dichotomous outcomes were analysed by calculating Peto odds ratios (OR) and 95% confidence intervals (95% CI). If there were sufficient homogeneities among the included studies, meta-analyses were performed. Review Manager Version 5.2 (The Nordic Cochrane Centre, The Cochrane Collaboration, 2012) was used to conduct this analysis.

## Results

### Study selection

**Fig 1:**
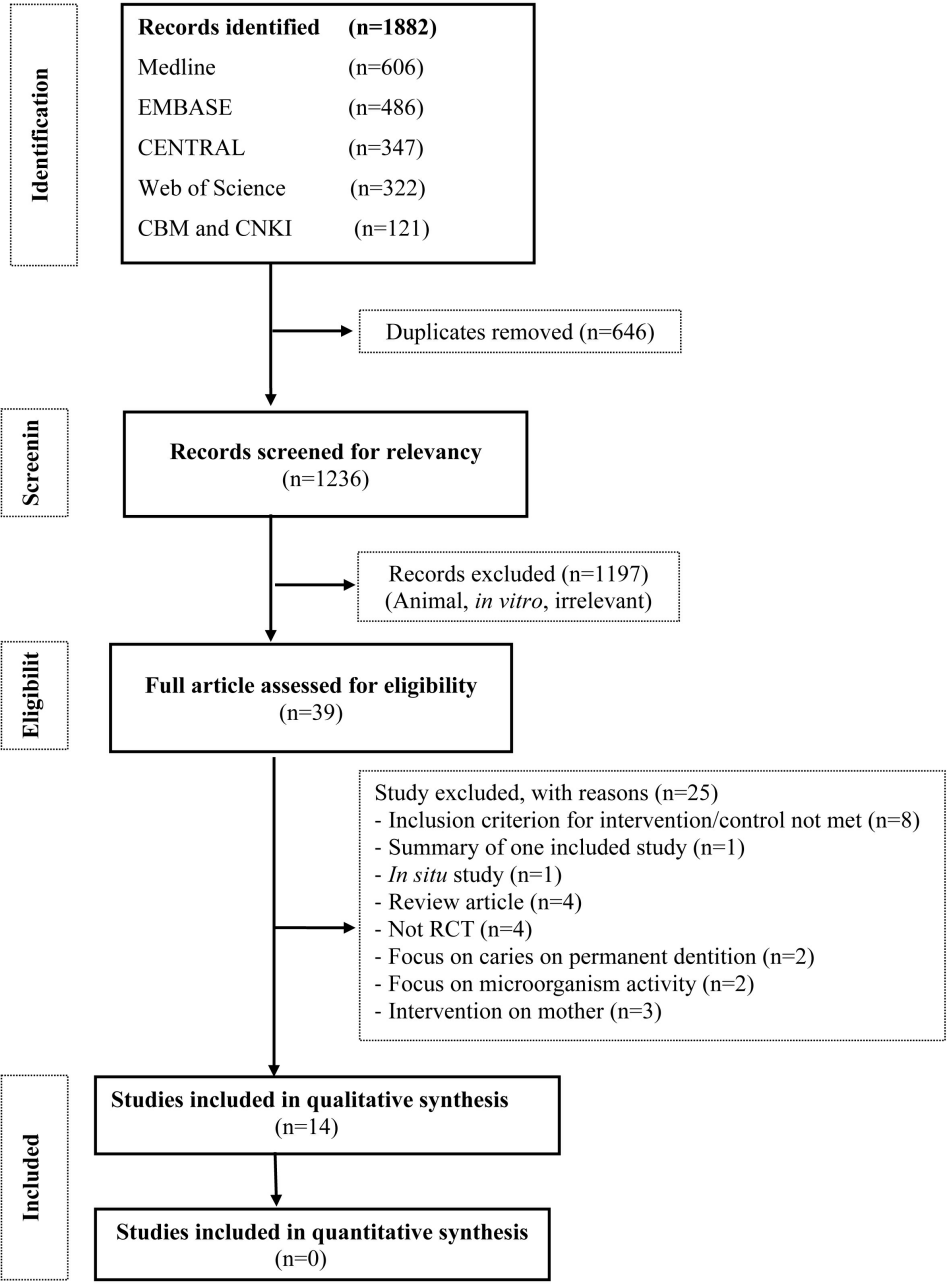
Systematic review flow diagram. (RCT = Randomized controlled trials).

A total of 1,236 articles were screened for relevance (Fig 1). After applying inclusion and exclusion criteria, 1,197 studies did not meet the inclusion criteria and were excluded. A total of 39 full papers were retrieved and reviewed, among which 25 articles were excluded for the reasons given in S2 Table. Finally, 14 studies [19–32] were included in the systematic review.

### Study characteristics

In the 14 articles included in the review (Table 1), a total of 4,269 participants were evaluated, all of them were classified as healthy by the authors. All studies reported the age of participants (age range, 0 to 11 years), the sample size (ranged from 37 to 1,306), and the duration of the study (varied from 3 to 36 months). The delivery modalities of the agents included in the systematic review were sugarless mint confection, paste, gel, varnish, snacks, tablets and wipes (Table 1).

**Table 1:** Summary of the included studies.

Type of intervention	First Author (year)	Participants invention/control (age)	Follow-up protocol	Intervention modality	Comparative/control	Assessment method	Primary outcome
Arginine	Acevedo,AM. (2008)	96/99 subjects (10.5-11y)	Baseline, 6, 12m	Confection containing CaviStat^®^ + F toothpaste,4 times a day	Control confection without CaviStat^®^ + F toothpaste	defs scores	Test / control (Mean±SD)
							defs:
							0m: 0.67±1.66 / 0.51±1.21
							6m: 0.95±1.46 / 1.29±1.85
							12m: 0.81±1.43 / 1.09±1.57
Chlorhexidine	Gisselsson, H. (1994)	59/58/116 subjects (4-7y)	Baseline, 3y	(1)1% chlorhexidine gel,4 times a year	(1) Placebo gel,4 times a year	defs scores	Test/placebo/control (Mean±SD)
					(2) Control group (no treatment)		Baseline: 0.19/0.03/0.4
							3 years: 2.78±3.27/4.57±4.04/4.6±4.86
	Tai, B.J. (2003)	440/451/415 subjects (3-7y)	Baseline, 2y	40% chlorhexidine varnish, once every 6 months	(1) Placebo varnish, once every 6 month	defs scores/dmfs molar scores	Test/placebo/control (Mean±SD)
					(2) No treatment		3yrs group:
							Baseline: 2.98±4.72/4.20±5.86/4.6±4.86
							2 years: 4.20±5.86/4.64±5.45/4.11±5.68
							5yrs group:
							Baseline: 2.81±4.22/2.63±4.14/3.07±4.23
							2 years: 3.81±4.58/4.24±4.93/4.9±5.2
							6-7yrs group:
							Baseline: 0.25±0.75/0.41±0.85/0.3±0.93
							2 years: 0.52±1.41/0.9±1.28/1.77±1.44
	Baca, P. (2004)	86/95 subjects (6-7y)	Baseline, 2y	(1) 1% chlorhexidine-thymol varnish, once every 3 months	Control group (no treatment)	dmft and dmfs scores	Test/ control (Mean±SD)
							dmfs:
							Baseline: 3.75±6.75/3.07±6.03
							2y:(dft = 0 at baseline) 0.94±2.10/1.73±2.68
							(dft>0 at baseline) 3.04±3.15/3.4±3.88
	Du, M.Q. (2006)	155/135 subjects (4-5y)	Baseline, 2y	40% chlorhexidine varnish, once every 6 months	Placebo varnish, once every 6 month	dmfs molar scores	Test/control (Mean±SD)
							dmfs-molar:
							Baseline: 2.8±0.3/2.6±0.4
							2y:3.8±0.4/4.2±0.4
	Amorim, R.G. (2008)	18/19/20/19 subjects (3-5y)	Baseline, 3m	(1) Chlorhexidine varnish	Control group (no treatment)	Clinical assessment was performed at 1 m and 3 m follow-up with VPI and WS scores	Test1/test 2/test 3/control (Mean ±SD)
				(2) Fluoride varnish			WS score (T1-T3):
				(3) Chlorhexidine and fluoride varnish once every week,4 times.			-0.89±1.45/-1.05±1.54/-1.4±2.21 / 0.37±1.01
	Plonka, K.A (2013)	183/171/188 subjects (6-24m)	6 m (baseline)/12 /18 /24 m	(1) Twice daily tooth-brushing+ 0.12% chlorhexidine gel, once daily	No product+ twice daily tooth-brushing	Percentage of children with ECC/ MS/LB	Test 1/test 2/control
				(2) Twice daily tooth-brushing+ 10% CPP-ACP paste, once daily			Number with caries/Total (%)
							Test 1: 4/180 (2%)
							Test 2: 2/163 (1%)
							Control: 3/188 (2%)
	Pukallus, M.L. (2013)	61/58subjects(0-2y)	Newborn, 6/12/18/24 m	Twice daily low-dose fluoride toothpaste+ 0.12% chlorhexidine gel, once daily	Low-dose fluoride toothpaste tooth-brushing, twice daily	Percentage of children with ECC/ MS/LB	Test/control:
							Number with caries/Total (%)
							Test: 3/61 (5%)
							Control: 4/58 (7%)
							Children with MS: 28(46%)/27(47%)
							Children with LB: 116(63%)/38(66%)
CPP-ACP	Sitthisettapong, T. (2012)	117/112 subjects (2.5–3.5y)	Baseline, 6m, 1y	10% CPP-ACP mousse + fluoride toothpaste	Placebo toothpaste + fluoride toothpaste	Clinical assessmentwith ICDAS criteria	Test 1/ test 2/ control (Mean±SD)
							Baselineto12month (No progression/ progression): OR: 1.002, 95%CI (0.86,1.17)
	Memarpor, M. (2015)	30/29/31/32 subjects (1-3y)	Baseline, 4,8,12m	(1) Oral hygiene and CPP-ACP mousse, twice a day	Control (no treatment)	Change in mean WSL size and change in dmft index	Test1/teat 2/control 1/control 2
				(2) Oral hygiene and 5% NaF vanish			Baseline to 12 m: -0.63±0.62/-0.51±10.56/-0.1±1.12/1.15±1.26
				(3) Oral hygiene +diet counselling			Dmft index:
							12 m: 0.17±0.53/0.3±0.9/0.42±0.99/2±2
Triclosan	Cao, H.Z. (2007)	296/265 subjects (2–5 y)	Baseline, 1 y	0.3% triclosan vanish, twice a year	Control (no treatment)	Dmft and dmfs scores	Test/control (Mean ±SD)
							Dmft
							Baseline: 0.98±2.11/0.93±2.06
							12m: 1.34±2.60/1.72±2.82
							dmfs
							Baseline: 1.24±2.60/1.18±2.28
							12m: 1.47±2.54/2.11±2.93
Xylitol	Oscarson, P. (2006)	55/63subjects(2-4y)	Baseline, 24m	Xylitol tablet, once per day. Two tablet per day after 6 m. This intervention was terminated when children was 3.5 yrs old	No tablet	defs score and salivary MS level	Test/control
							Baseline: Number with caries/total (%)
							Test: 4/66 (6%)
							Control: 4/55 (7%)
							24m dmfs: 0.8±2.8 / 1.2±3.5
	Zhan, L. (2012)	20/17subjects(6-35m)	Baseline, 3,6,12m	Xylitol wipe,6 wipes daily+ tooth brushing	Placebo wipe	defs score and salivary MS and LB level	Number with caries/Total (%)
							Test: 1/20 (5%)
							Control: 6/17 (35%)
							MS level (Test/control):
							Baseline: 1.25±2.28/1.1±2.0
							1yr: 1.25±2.63/3.38±2.75
							LB level (Test/control):
							Baseline: 0.14±0.65/0.07±0.34
							1yr: 0.11±0.48/0.53±1.21
	Lee, W. (2015)	122/138 subjects (5-6y)	Baseline, 30m	Xylitol gummy bear, 3 times per day	Placebo gummy bear	dmfs score	Test/control
							New d3-6mfs: 5.0±7.6/4.0±6.5
							New d1-6mfs: 5.7±7.6/4.7±6.7

CaviStat®: an arginine bicarbonate calcium carbonate complex; WS: white spot; WSL: white spot lesion; F:fluoride; defs: decayed, extracted, filled surfaces; dmfs: decayed, missing, filled surfaces; dmft: decayed, missing, filled tooth; VPI:Visible Plaque Index; ECC: Early Childhood Caries; MS: mutans streptococci; LB: lactobacilli; ICDAS:The International Caries Detection and Assessment System; m: month; y:year.

One study reported the topical application of arginine for the prevention of dental caries on primary molars [19]. Seven studies investigated the efficacy of chlorhexidine on the prevention of caries in primary dentition compared with other interventions [20–23,25,30,31]. Two studies compared the effect of daily application of CPP-ACP on dental caries in preschool children with fluoride varnish or placebo [24,26]. Three studies reported the anti-caries effect of xylitol in the form of wipes, tablets and snacks on caries progression in primary teeth [27–29]. One study assessed the clinical effect of the 0.3% triclosan varnish on caries prevention [32].

### Primary outcome of the studies

The primary outcome measures used in the 14 studies included clinical assessment using the International Caries Detection and Assessment System (ICDAS) criteria or dmfs/dmft index, clinical assessment for the white spot lesions and VPI (Visible Plaque Index), and bitewing radiography for approximal caries increment. No study used fluorescence-based assessments.

One study found arginine-containing mint confection significantly reduced caries development in primary molars compared with placebo after 6 months and 12 months [19].

Seven studies showed that chlorhexidine had caries-reducing potential in primary teeth [19–23,25,30,31]. However, there was conflicting evidence regarding the efficacy of chlorhexidine when used in conjunction with fluoride. One study found the combined application of chlorhexidine and low-dose fluoride did not significantly increase the caries-preventive effect compared with low-dose fluoride alone [20]. Another two studies reported the synergistic effect of chlorhexidine with fluoride in controlling the demineralization of primary dentition [21,23].

One study reported that the application of CPP-ACP mousse during the 12-month intervention period reduced the size of white spot lesions in the anterior primary teeth and was associated with smaller increase in dmft index values compared with placebo or fluoride varnish [24]. However, another study did not find the daily use of CPP-ACP paste was any better at controlling early childhood caries than low-fluoride toothpaste [20]. In addition, the daily application of 10% w/v CPP-ACP and fluoride toothpaste did not show synergism in preventing caries in the primary dentition of pre-school children [26].

Xylitol wipes (4.2g/d) were found to be effective in controlling dental caries in primary teeth [29]. However, a study that evaluated products containing low-doses of xylitol (0.5–1.0 g/tablet [27]) did not find any cariostatic activity. The daily application of xylitol gummy bears (7.8 g/d) did not provide additional benefit beyond regular oral hygiene [28].

One study indicated that 0.3% triclosan varnish was more effective than blank control in reducing caries incidence for children aged 2–5 years old during its one-year follow-up period [32].

### Secondary outcomes of the studies

Some studies included in the systematic review failed to report on the side effects of these non-fluoride agents [19,21,22,23,27,32]. Four studies presented information on the side effects of chlorhexidine varnish and gel. No serious side effects were reported during the 24-month observation period [20, 25, 30,31]. The use of 10% CPP-ACP paste was also found to have no adverse effects [24]. Another study of CPP-ACP paste failed to provide information on side effects [26]; the author was contacted by email and confirmed that no extra calculus formation had occurred on the primary teeth in their experimental group. In a 12-month trial of xylitol wipes, no side effects (including allergy, flatulence and diarrhoea) were reported by the parents [29]. No major side effects from the xylitol-containing gummy bears intervention was reported [28].

### Risk of bias

**Fig 2:**
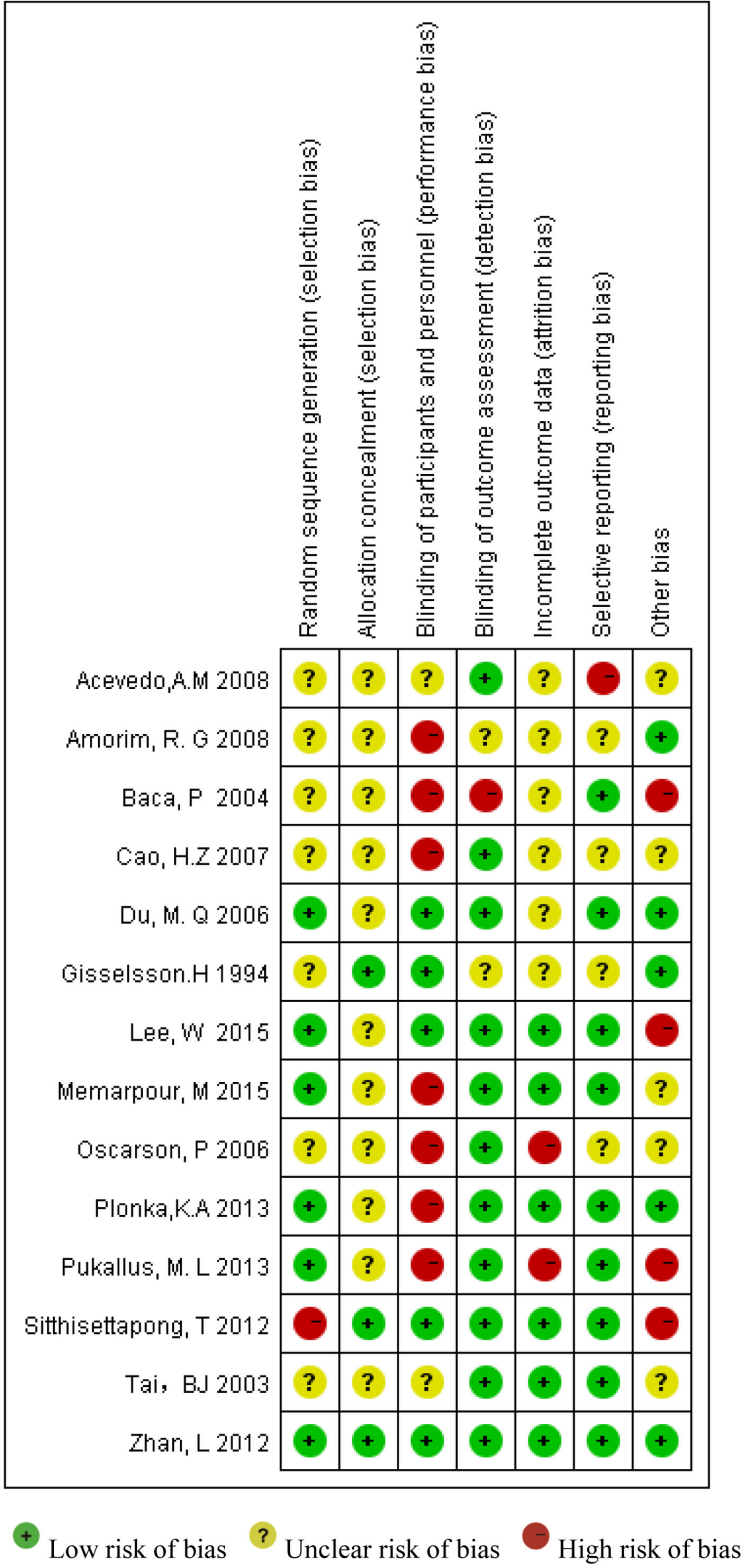
Risk of bias summary.

**Fig 3:**
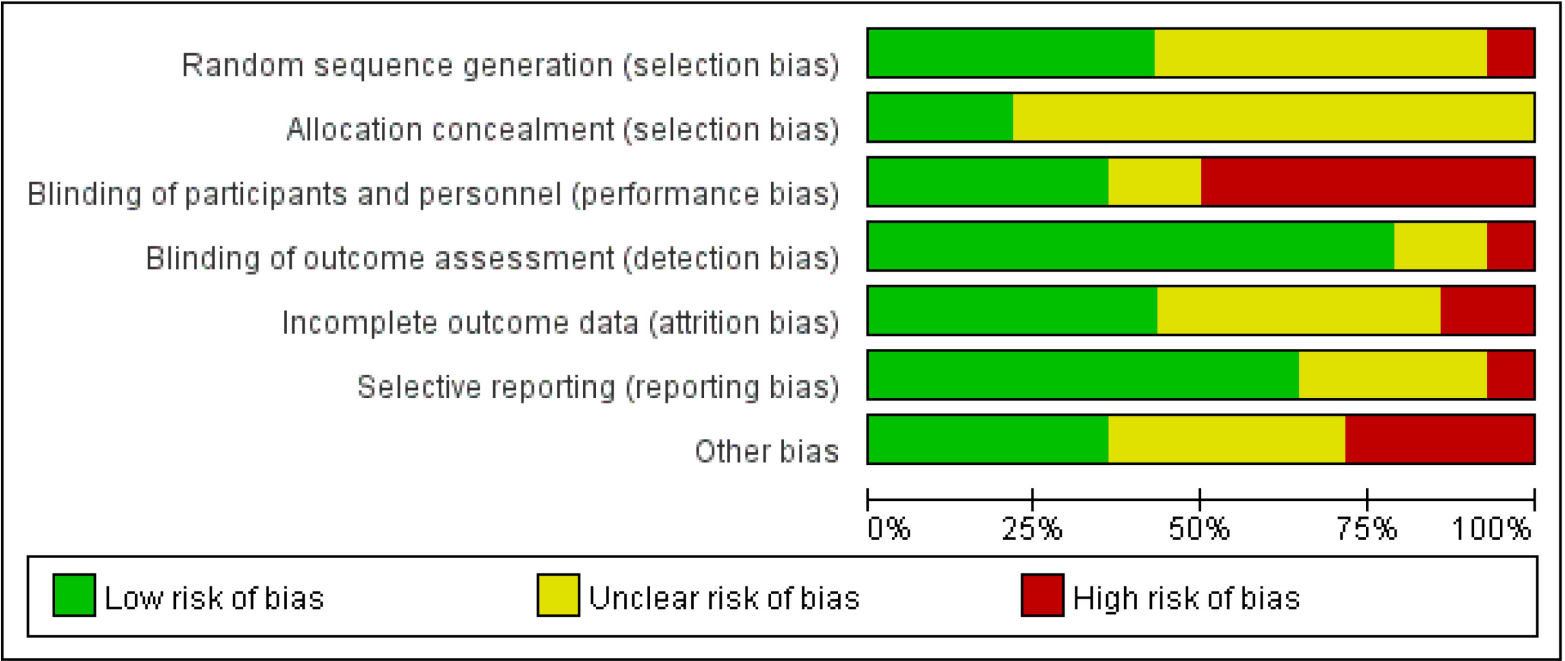
Levels of bias types.

The risk of bias assessment revealed that only one study had a low risk of bias [29], three studies had an unclear risk of bias [23,30,31], and the remaining 10 were scored as high risk of bias [19–22,24–28,32] (Figs 2 and 3).

## Discussion

The current review systematically assessed the effect of non-fluoride agents compared with placebos and/or fluoride in the prevention of caries in primary dentition. Five non-fluoride agents were used in the included studies: arginine, chlorhexidine, CPP-ACP, triclosan and xylitol. The current research evidence is not sufficient to confirm that the use of these non-fluoride agents is more effective than placebo or fluoride for preventing dental caries in primary dentition.

One limitation of this systematic review is that the general quality of the currently available RCTs was not high. Of the 14 studies included in this review, only one study was assessed as having low risk of bias [29], and the others were graded either unclear or at high risk of bias. The most common risks of bias were selection bias and detection bias. Future trials should adopt a parallel group design that incorporates the use of randomization, blinding, as well as the allocation concealment method.

Meta-analyses are usually performed for studies with similar interventions and outcome measures. In this review, two studies on the effect of chlorhexidine varnish on dental caries in preschool children [30,31] were found to be homogeneous, however, they were conducted by the same group of authors and on the same patient population. Thus meta-analyses could not be carried out due to publication bias.

Another important concern is that some of the studies included in the review did not present information concerning adverse effects [19,21,22,23,27,32]. For example, xylitol has been found to cause side effects such as bloating, flatulence and diarrhoea [17]; chlorhexidine sometimes causes staining of the teeth and tongue, mucosal soreness and desquamation, temporary taste disturbances, parotid gland swelling and hypersensitivity [33,34]. Thus safety assessments should be considered as an essential part of a well-designed RCT.

Arginine-containing products have been found to provide significant benefits in managing dental caries in previous studies [35,36]. In this review, an RCT investigating CaviStat [19], which contains arginine, and which was incorporated into a sugarless mint confection, appeared to be effective in reducing caries development and progression in primary teeth. However, this was the only study in this review to meet the inclusion and exclusion criteria and this study was scored as having high risk of bias. Thus, well-designed clinical studies are still required to provide adequate evidence on the effect of arginine on primary dentition.

Fluoride is generally known to promote remineralization, but the use of fluoride alone has been reported to be insufficient to prevent progressive mineral loss [1]. Chlorhexidine, a cationic bis-biguanide with a broad spectrum of antibacterial activity, has been well recognized as a chemotherapeutic agent active against *Streptococcus mutans*, which plays a major role in tooth decay [16]. Several clinical trials and *in vitro* studies have demonstrated that combination treatment with chlorhexidine and fluoride is effective in caries reduction [37–41]. However, the synergistic effect of chlorhexidine and fluoride is still unclear [25]. This synergistic effect between chlorhexidine and fluoride were not supported by all clinical trials[20],although in Plonka’s study[20] the caries rate in the intervention and control groups were both very low (For details, see Table 1).

Numerous studies have demonstrated the anti-caries effect of CPP-ACP by promoting remineralization and inhibiting demineralization [42,43]. They also reported that CPP-ACP was effective in repairing the microstructure of enamel through significantly increased hydroxyapatite crystal size and calcium/ phosphorus mol ratios. The result is in agreement with a recent review, which indicated a long-term remineralization efficacy of CPP-ACP on carious lesions [44]. The advantage of using CPP-ACP as the agent rather than fluoride, however, is still unclear. Since fluoride-containing toothpastes are now widely used for daily oral hygiene, it is recommended that future RCTs consider using fluoride as a control.

As a non-cariogenic sweetener, the cariostatic effect of xylitol on primary dentition has been investigated in several clinical studies [45,46]. Its anti-caries effect remains controversial according to this review. One reason for this may be the dose-effect relationship. It has been found that exposure to at least 4g xylitol/day is necessary to be clinically effective [47,48] The low xylitol dose (0.5-1g/tablet), which was used in one study [27] included in the review, did not prevent caries in primary teeth. Other reasons for the inconclusive results are the limited sample sizes. The study that evaluated the effects of xylitol-containing wipes found that the daily application of xylitol (4.2g/day) was useful in controlling caries in preschool children [29], however, the sample size was 20 or fewer for each group, hence, the conclusion should be interpreted with caution.

The effectiveness of administering these non-fluoride agents, *e*.*g*. daily topical toothpaste, may be influenced by patient compliance, especially in children who usually show relatively poor cooperation compared to adults [49]. Delivery vehicles that are less patient-dependent, such as varnishes that are applied by professionals, have been shown to be effective in children [13,22,24,30]. In addition to the therapeutic agents included in the review, other non-fluoride agents, such as bioactive glass, pit and fissure sealants and resin infiltration, have also been extensively studied [50–52]. However, those studies did not meet the inclusion criteria for this review, because most of them either focused on the caries-preventive effect on permanent teeth or were not RCTs. High quality RCTs on the preventive effect of non-fluoride agents in primary dentition are still required.

The follow-up period of the studies included in the review varied from 3 to 36 months. Although the duration of the clinical trials involving cariostatic products should, according to the consensus statement by the International Consensus Workshop on Caries Clinical Trials [53], be sufficient to observe the progression of carious lesion development (2 to 3 years),the participants in the studies on the preventive measures for dental caries in primary dentition were all children; and mostly they were caries-active. Considering that primary teeth are more prone to carious lesion progression, the observation period could be relatively shorter for primary dentition than for permanent dentition. Previous studies have demonstrated a follow-up period should be for at least three months [42,44]. However, studies show that the anti-caries effects of non-fluoride agents were not stable over time. It has been found that using chlorhexidine-containing varnishes resulted in early reductions in mutans streptococci (MS) levels at 3 and 6 months in the permanent dentition, but this effect was not sustained after longer periods of follow-up at 12, 24 and 36 months [54]. Similarly, another study also showed no reduction in the MS level in mothers after xylitol-gum use for 2 years [55]. These results could be due to the development of MS resistance to chlorhexidine/xylitol over time. Although the MS level is a proxy outcome, it is unclear how reductions would translate into any effect on caries prevention. Considering the influence of time, an adequate follow-up period should be employed to reveal all the potential effects, both beneficial and detrimental, of the intervention method.

A number of non-invasive methods have been developed as potential diagnostic aids for clinicians, for example, quantitative light-induced fluorescence (QLF), a system based on the measurement of fluorescence loss following enamel demineralization [56]. This method has shown high sensitivity and specificity in detecting caries lesions [57,58,59]. Optical Coherence Tomography (OCT) is another non-invasive imaging technique that constructs high-resolution cross-sectional images of internal biological structures [60]. Previous studies have shown that OCT has the potential to detect, and quantify, demineralization in *in vitro* caries-like models [61,62].Photothermal radiometry and modulated luminescence (PTR/LUM), commercially marketed as the Canary System^®^ (Quantum Dental Technologies, Toronto, Ont.,Canada) is based on the combination of two slightly different responses of the tooth tissues from a periodic irradiation with a pulsating laser beam [63].The Canary System has demonstrated a greater accuracy in detecting proximal lesions than International Caries Detection and Assessment System (ICDAS) II and bitewing radiography (BW) [64]. Moreover, the Canary System also serves as a clinical tool to detect and monitor the status of caries lesions and tooth structure underneath sealant [65]. These new methods serve as supplemental aids to traditional visual examination (*e*.*g*, the World Health Organization method) and have been generally considered to have high accuracy but low repeatability between different examiners [66]. However, all the stuies included in this review used only traditional methods for detecting and monitoring dental caries. It would be beneficial in future studies to use a combination of the traditional and electronic diagnosis methods to investigate the initiation, progression and reversal of dental caries [66].

## Conclusion

A study at low risk of bias indicated that daily use of xylitol wipes is a useful adjunct for caries control in young children; however, this conclusion should be interpreted with caution as this study had a very limited sample size (20/17). Chlorhexidine and CPP-ACP may be more effective than placebo in managing caries in primary dentition, but their efficacy relative to fluoride is still unclear. Arginine-containing mint confection and 0.3% triclosan varnish were found to reduce caries development in primary teeth but the evidence was at high risk of bias. High quality randomized controlled trials are needed to make a definitive recommendation.

## Supporting information

S1 File Search strategies. Table A in S1 File. Search strategy for Medline via PubMed. Table B in S1 File. Search strategy for EMBASE. Table C in S1 File. Search strategy for Cochrane library. Table D in S1 File. Search strategy for Web of Science. Table E in S1 File. Search strategy for CBM. Table F in S1 File. Search strategy for CNKI.
(DOCX)

S1 Table PRISMA 2009 checklist. (DOCX)

S2 Table Articles excluded from this review. (DOCX)

S3 Table Risk of bias in included studies. (DOCX)

## References

[1] Rošin-Grget K, Peroš K, Sutej I, Bašić K. The cariostatic mechanisms of fluoride. Acta Med Acad. 2013; 42:179–188. doi: 10.5644/ama2006-124.85 24308397 10.5644/ama2006-124.85

[2] Murray JJ, Rugg-Gunn AJ, Jenkins GN, editors. Water fluoridation and child dental health. In: Fluorides in Caries Prevention. Mumbai: Varghese; 1999. p 39–63.

[3] Dye BA, Tan S, Smith V, Lewis BG, Barker LK, Thornton-Evans G, et al Trends in oral health status: United States, 1988–1994 and 1999–2004. Vital and health statistics. Series 11, Data from the national health survey, 2007; 248: 1–92. 17633507

[4] Al-Malik MI, Holt RD, Bedi R, Speight PM. Investigation of an index to measure tooth wear in primary teeth. J Dent. 2001;29: 103–107. 11239584 10.1016/s0300-5712(00)00064-6

[5] Johansson AK, Sorvari R, Birkhed D, Meurman JH. Dental erosion in deciduous teeth—an in vivo and in vitro study. J Dent. 2001;29: 333–340. 11472805 10.1016/s0300-5712(01)00029-x

[6] Lynch E, Baysan A. Reversal of primary root caries using a dentifrice with a high fluoride content. Caries Res. 2001;35: 60–64. 11359061 10.1159/000049113

[7] Featherstone JD. Prevention and reversal of dental caries: role of low level fluoride. Community Dental Oral Epidemiol. 1999;27: 31–40 10.1111/j.1600-0528.1999.tb01989.x 10086924

[8] Dennison JB, Straffon LH, Smith RC. Effectiveness of sealant treatment over five years in an insured population. J Am Dent Assoc. 2000;131: 597–605. 10832253 10.14219/jada.archive.2000.0233

[9] Alanen P, Isokangas P, Gutmann K. Xylitol candies in caries prevention: results of a field study in Estonian children. Community Dent Oral Epidemiol. 2000;28: 218–224 10830649 10.1034/j.1600-0528.2000.280308.x

[10] Emilson CG. Potential efficacy of chlorhexidine against mutans streptococci and human dental caries. Dent Res. 1994;73: 682–691. 10.1177/00220345940730031401 8163738

[11] Wang Y, Mei L, Gong L, Li J, He S, Ji Y, et al Remineralization of early enamel caries lesions using different bioactive elements containing toothpastes: An in vitro study. Technol Health Care. 2016; 14; 24:701–11 doi: 10.3233/THC-161221 27233091 10.3233/THC-161221

[12] Marinho VC, Higgins JP, Sheiham A, Logan S. Combinations of topical fluoride (toothpastes, mouthrinses, gels, varnishes) versus single topical fluoride for preventing dental caries in children and adolescents. Cochrane Database Syst Rev. 1:CD002781. 10.1002/14651858.CD002781.pub2 PMC6999808 14973992

[13] Marinho VC, Higgins JP, Logan S, Sheiham A. Topical fluoride (toothpastes, mouthrinses, gels or varnishes) for preventing dental caries in children and adolescents. Cochrane Database Syst Rev. 2003; 4:CD002782. 10.1002/14651858.CD002782 PMC6999805 14583954

[14] Marinho VC, Higgins JP, Sheiham A, Logan S. One topical fluoride (toothpastes, or mouthrinses, or gels, or varnishes) versus another for preventing dental caries in children and adolescents. Cochrane Database Syst Rev.2004;1:CD002780. 10.1002/14651858.CD002780.pub2 PMC6999809 14973991

[15] Lagerweij MD, Ten Cate JM. Remineralization of enamel lesions with daily applications of a high-concentration fluoride gel and a fluoridated toothpaste: An in situ study. Caries Res.2002;36: 270–274. 12218276 10.1159/000063929

[16] Walsh T, Oliveira-Neto JM, Moore D. Chlorhexidine treatment for the prevention of dental caries in children and adolescents. Cochrane Database Syst Rev. 2015; 4:CD008457. 10.1002/14651858.CD008457.pub2 PMC10726983 25867816

[17] Riley P, Moore D, Ahmed F, Sharif MO, Worthington HV. Xylitol-containing products for preventing dental caries in children and adults. Cochrane Database Syst Rev. 2015;26:CD010743. 10.1002/14651858.CD010743.pub2 PMC9345289 25809586

[18] Rethman MP, Beltrán-Aguilar ED, Billings RJ, Hujoel PP, Katz BP, Milgrom P, Sohn W, et al Aravamudhan K, Frantsve-Hawley J, Meyer DM; American Dental Association Council on ScientificAffairs Expert Panel on Nonfluoride Caries-Preventive Agents. Nonfluoridecaries-preventive agents: executive summary of evidence-based clinicalrecommendations. J Am Dent Assoc. 2011;142:1065–1071. 21987836 10.14219/jada.archive.2011.0329

[19] Acevedo AM, Montero M, Rojas-Sanchez F, Machado C, Rivera LE, Wolff M, et al Clinical evaluation of the ability of CaviStat® in a mint confection to inhibit the development of dental caries in children. J Clin Dent. 2008;19: 1–8. 18500152

[20] Plonka KA, Pukallus ML, Holcombe TF, Barnett AG, Walsh LJ, Seow WK, et al Randomized controlled trial: a randomized controlled clinical trial comparing a remineralizing paste with an antibacterial gel to prevent early childhood caries. Pediatr Dent. 2013;35: 8–12. 23635884

[21] Amorim RG, Leal SC, Bezerra AC, Amorim FP, Toledo OA. Association of chlorhexidine and fluoride for plaque control and white spot lesion remineralization in primary dentition. Int J Paediatr Dent. 2008;18: 446–51. doi: 10.1111/j.1365-263X.2008.00914.x 18489576 10.1111/j.1365-263X.2008.00914.x

[22] Baca P, Muñoz MJ, Bravo M, Junco P, Baca AP. Effectiveness of chlorhexidine-thymol varnish in preventing caries lesions in primary molars. J Dent Child (Chic).2004;71: 61–65. 15272659

[23] Gisselsson H, Birkhed D, Björn AL.Effect of a 3-year professional flossing program with chlorhexidine gel on approximal caries and cost of treatment in preschool children. Caries Res.1994;28: 394–399. 8001065 10.1159/000262008

[24] Memarpour M, Fakhraei E, Dadaein S, Vossoughi M. Efficacy of fluoride varnish and casein phosphopeptide-amorphous calcium phosphate for remineralization of primary teeth: a randomized clinical trial. Med Princ Pract 2015;24: 231–237. doi: 10.1159/000379750 25895964 10.1159/000379750 PMC5588292

[25] Pukallus ML, Plonka KA, Barnett AG, Walsh LJ, Holcombe TF, Seow WK. A randomised, controlled clinical trial comparing chlorhexidine gel and low-dose fluoride toothpaste to prevent early childhood caries. Int J Paediatr Dent. 2013;23: 216–224. doi: 10.1111/j.1365-263X.2012.01248.x 22713081 10.1111/j.1365-263X.2012.01248.x

[26] Sitthisettapong T, Phantumvanit P, Huebner C, De Rouen T. Effect of CPP-ACP Paste on Dental Caries in Primary Teeth: A Randomized Trial. Dent Res. 2012;91:847–852. doi: 10.1177/0022034512454296 22805294 10.1177/0022034512454296 PMC3420390

[27] Oscarson P, Lif Holgerson P, Sjöström I, Twetman S, Stecksén-Blicks C. Influence of a low xylitol-dose on mutans streptococci colonisation and caries development in preschool children. Eur Arch Paediatr Dent.2006;7: 142–147. 17140543 10.1007/BF03262555

[28] Lee W, Spiekerman C, Heima M, Eggertsson H, Ferretti G, Milgrom P, et al The effectiveness of xylitol in a school-based cluster-randomized clinical trial. Caries Res.2015;49: 41–49. doi: 10.1159/000360869 25428785 10.1159/000360869

[29] Zhan L, Cheng J, Chang P, Ngo M, Denbesten PK, Hoover CI, et al Effects of xylitol wipes on cariogenic bacteria and caries in young children. J Dent Res. 2012;91: 85–90. 10.1177/0022034511434354 PMC3383105 22699675

[30] Du MQ, Tai BJ, Jiang H, Lo EC, Fan MW, Bian Z. A two-year randomized clinical trial of chlorhexidine varnish on dental caries in Chinese preschool children. J Dent Res. 2006;85: 557–559. doi: 10.1177/154405910608500615 16723655 10.1177/154405910608500615

[31] Tai BJ, Jiang H, Du MQ. A two-year study of 40% chlorhexidine varnish on the prevention of dental caries. J Oral Sci Res.2003;19: 521–523.

[32] Cao HZ, Wang S, Pan Y. An investigation of the clinical effect of the 0.3% Triclosan varnish on caries prevention of primary teeth. Shanghai Kou Qiang Yi Xue. 2007;16: 8–10. 17377691

[33] British Medical Association and Royal Pharmaceutical Society. British National Formulary. Vol.Jan 2015, London: BMJ Publishing Group Ltd and Royal Pharmaceutical Society, 2015.

[34] Krishna MT, York M, Chin T, Gnanakumaran G, Heslegrave J, Derbridge C, et al Multi-centre retrospective analysis of anaphylaxis during general anaesthesia in the United Kingdom: aetiology and diagnostic performance of acute serum tryptase. Clin Exp Immunol. 2014;178: 399–404. doi: 10.1111/cei.12424 25070464 10.1111/cei.12424 PMC4233389

[35] Hu DY, Yin W, Li X, Feng Y, Zhang YP, Cummins D, et al A clinical investigation of the efficacy of a dentifrice containing 1.5% arginine and 1,450 ppm fluoride, as sodium monofluorophosphate in a calcium base, on primary root caries. J Clin Dent. 2013;24: A23–A31. 24156137

[36] Srisilapanan P, Korwanich N, Yin W, Chuensuwonkul C, Mateo LR, Zhang YP, et al Comparison of the efficacy of dentifrice containing 1.5% arginine and 1,450 ppm fluoride to a dentifrice containing 1,450 ppm fluoride alone in the management of early coronal caries as assessed using quantitative light-induced fluorescence. J Dent. 2013;41: S29–S34 doi: 10.1016/j.jdent.2010.04.005 23985436 10.1016/j.jdent.2010.04.005

[37] Schaeken MJ, De Haan P. Effects of sustained-release chlorhexidine acetate on the human dental plaque flora. J Dent Res. 1989;68: 119–23. doi: 10.1177/00220345890680020401 2918132 10.1177/00220345890680020401

[38] Matthijs S, Adriaens PA. Chlorhexidine varnishes: a review. Journal of Clinical Periodontology. 2002; 29:1–8. 10.1034/j.1600-051x.2002.290101.x 11846842

[39] Rodrigues CR, Marquezan M, Barroso LP, Grande RHM, Myaki SI, Kabakura V, et al Effect of chlorhexidine-thymol varnish on caries lesion development in first permanent molars. J Clin Dent. 2008;19: 18–21. 18500155

[40] Katz S. The use of fluoride and chlorhexidine for the prevention of radiation caries. J Am Dent Assoc. 1982;104: 164–170. 6948892 10.14219/jada.archive.1982.0016

[41] Emilson CG. Potential efficacy of chlorhexidine against mutans streptococci and human dental caries. J Dent Res. 1994;73: 682–91 doi: 10.1177/00220345940730031401 8163738 10.1177/00220345940730031401

[42] Zhou C, Zhang D, Bai Y, Li S. Casein phosphopeptide- amorphous calcium phosphate remineralization of primary teeth early enamel lesions. J Dent. 2014;42: 21–29. doi: 10.1016/j.jdent.2013.11.005 24269831 10.1016/j.jdent.2013.11.005

[43] Rao SK, Bhat GS, Aradhya S, Devi A, Bhat M. Study of the efficacy of toothpaste containing casein phosphopeptide in the prevention of dental caries: a randomized controlled trial in 12- to 15-year-old high caries risk children in Bangalore, India. Caries Res. 2009;43: 430–5. doi: 10.1159/000252976 19864905 10.1159/000252976

[44] Li J, Xie X, Wang Y, Yin W, Antoun JS, Farella M_,_ et al Long-term remineralizing effect of casein phosphopeptide-amorphous calcium phosphate (CPP-ACP) on early caries lesions in vivo: a systematic review. J Dent. 2014;42: 769–777. doi: 10.1016/j.jdent.2014.03.015 24705069 10.1016/j.jdent.2014.03.015

[45] Alanen P, Isokangas P, Gutmann K. Xylitol candies in caries prevention: results of a field study in Estonian children. Community Dent Oral Epidemiol. 2000; 28:218–24. 10830649 10.1034/j.1600-0528.2000.280308.x

[46] Hujoel PP, Mäkinen KK, Bennett CA, Isotupa KP, Isokangas PJ, Allen P, et al The optimum time to initiate habitual xylitol gum chewing for obtaining long-term caries prevention. J Dent Res. 1999; 78:797–803. doi: 10.1177/00220345990780031301 10096456 10.1177/00220345990780031301

[47] Maguire A, Rugg-Gunn AJ. Xylitol and caries prevention–is it a magic bullet? Br Dent J. 2003;194: 429–436. doi: 10.1038/sj.bdj.4810022 12778091 10.1038/sj.bdj.4810022

[48] Lif Holgerson P, Stecksén-Blicks C, Sjöström I, Twetman S. Effects of xylitol containing chewing gums on interdental plaque-pH in habitual xylitol consumers. Acta Odontol Scand. 2005; 63: 233–238. doi: 10.1080/00016350510019883 16040446 10.1080/00016350510019883

[49] Harris R, Nicoll AD, Adair PM, Pine CM. Risk factors for dental caries in young children: a systematic review of the literature. Community Dent Health. 2004;21: 71–85. 15072476

[50] Xu YT, Wu Q, Chen YM, Smales RJ, Shi SY, Wang MT. Antimicrobial effects of a bioactive glass combined with fluoride or triclosan on Streptococcus mutans biofilm. Arch Oral Biol. 2015;60: 1059–1065 doi: 10.1016/j.archoralbio.2015.03.007 25951616 10.1016/j.archoralbio.2015.03.007

[51] Meyer-Lueckel H, Bitter K, Paris S. Randomized controlled clinical trial on proximal caries infiltration: three-year follow-up. Caries Res. 2012;46: 544–548. doi: 10.1159/000341807 22922306 10.1159/000341807

[52] Tinanoff N, Coll JA, Dhar V, Maas WR, Chhibber S, Zokaei L. Evidence-based Update of Pediatric Dental Restorative Procedures: Preventive Strategies. J Clin Pediatr Dent. 2015; 39: 193–197. doi: 10.17796/1053-4628-39.3.193 26208061 10.17796/1053-4628-39.3.193

[53] Pitts NB, Stamm JW. International Consensus Workshop on Caries Clinical Trials (ICW-CCT)—final consensus statements: agreeing where the evidence leads. J Dent Res. 2004;83: C125–128. 15286139 10.1177/154405910408301s27

[54] Forgie AH, Paterson M, Pine CM, Pitts NB, Nugent ZJ. A randomised controlled trial of the caries-preventive efficacy of a chlorhexidine-containing varnish in high-caries-risk adolescents. Caries Res. 2000;34: 432–439. 11014911 10.1159/000016619

[55] Söderling E, Isokangas P, Pienihäkkinen K, Tenovuo J. Influence of maternal xylitol consumption on acquisition of mutans streptococci by infants. J Dent Res. 2000; 79:882–7. doi: 10.1177/00220345000790031601 10765964 10.1177/00220345000790031601

[56] Pretty IA, Ellwood RP. The caries continuum: opportunities to detect, treat and monitor the re-mineralization of early caries lesions. J Dent. 2013;41S: S12–21. 10.1016/j.jdent.2010.04.003 23985434

[57] De Josselin de Jong E, Sundstrom F, Westerling H, Tranaeus S, ten Bosch JJ, Angmar-Mansson B. A new method for in vivo quantification of changes in initial enamel caries with laser fluorescence. Caries Research. 1995;29: 2–7. 7867045 10.1159/000262032

[58] Angmar-Mansson B, ten Bosch JJ. Quantitative light-induced fluorescence (QLF): method for assessment of incipient caries lesions. Dentomaxillofacial Radiology. 2001;30: 298–307. doi: 10.1038/sj/dmfr/4600644 11641727 10.1038/sj/dmfr/4600644

[59] Ko HY, Kang SM, Kim HE, Kwon HK, Kim BI. Validation of quantitative light induced fluorescence-digital (QLF-D) for the detection of approximal caries in vitro. J Dent. 2015;43: 568–575. doi: 10.1016/j.jdent.2015.02.010 25724115 10.1016/j.jdent.2015.02.010

[60] Huang D, Swanson EA, Lin CP, Schuman JS, Stinson WG, Chang W, et al Optical coherence tomography. Science. 1991;254: 1178–1181. 1957169 10.1126/science.1957169 PMC4638169

[61] Wang XJ, Milner TE, de Boer JF, Zhang Y, Pashley DH, Nelson JS. Characterization of dentin and enamel by use of optical coherence tomography. Applied Optics. 1999;38: 2092–6. 18319769 10.1364/ao.38.002092

[62] Chew HP, Zakian CM, Pretty IA, Ellwood RP. Measuring initial enamel erosion with quantitative light-induced fluorescence and optical coherence tomography: an in vitro validation study. Caries Res. 2014;48: 254–62. doi: 10.1159/000354411 24481141 10.1159/000354411

[63] Wong B, Abrams SH, Tasevski C, Sivaguruthahan K, Silvertown J. Detection of interproximal caries in vitro using The Canary System. J Dent Res. 2014;93 (SpecIss A).

[64] Jan J, Wan Bakar WZ, Mathews SM, Okoye LO, Ehler BR, Louden C, et al Proximal caries lesion detection using the Canary Caries Detection System: an in vitro study. J Investig Clin Dent. 2016;7: 383–390. doi: 10.1111/jicd.12163 26012784 10.1111/jicd.12163

[65] Silvertown JD, Wong BP, Abrams SH, Sivagurunathan KS, Mathews SM, Amaechi BT. Comparison of The Canary System and DIAGNOdent for the in vitro detection of caries under opaque dental sealants. J Investig Clin Dent. 2016 9 26. doi:11.1111/jicd.12239. 10.1111/jicd.12239 27671372

[66] Gomez J. Detection and diagnosis of the early caries lesion. BMC Oral Health. 2015;15 Suppl 1:S3 doi: 10.1186/1472-6831-15-S1-S3 26392124 10.1186/1472-6831-15-S1-S3 PMC4580848

