# Supplementary material for: Effect of non-fluoride agents on the prevention of dental caries in primary dentition: A systematic review
Source: PLoS One. 2017 Aug 7;12(8):e0182221. doi: 10.1371/journal.pone.0182221 (PMC5546704; doi:10.1371/journal.pone.0182221)
Supplement: S1 File — Table A in S1 File. Search strategy for Medline via PubMed. Table B in S1 File. Search strategy for EMBASE. Table C in S1 File. Search strategy for Cochrane library. Table D in S1 File. Search strategy for Web of Science. Table E in S1 File. Search strategy for CBM. Table F in S1 File. Search strategy for CNKI. (DOCX) [file pone.0182221.s001.docx]

**S1 File. Search strategies**

**Table A in S1 File. Search strategy for Medline via PubMed**

#1 “tooth remineralization”[MeSH]

#2 “tooth demineralization”[MeSH]

#3 “dental caries”[MeSH]

#4 “white spot lesion” OR “white spot”[Title/Abstract]

#5 caries OR carious[Title/Abstract]

#6 #1 or #2 or #3 or #4 or #5 or #6

#7 “tooth, deciduous”[MeSH]

#8 “primary teeth” or “primary dentition*” or “primary tooth” or “deciduous tooth” or “deciduous teeth” or “milk teeth” or “milk tooth” or “baby tooth” or “baby teeth” [Title/Abstract]

#9 #7 or #8

#10 “randomized controlled trial”[pt]

#11 “controlled clinical trial” [pt]

#12 randomized [tiab]

#13 placebo [tiab]

#14 “clinical trials as topic” [MeSH:noexp]

#15 randomly [tiab]

#16 trial [tiab]

#17 #10 or #11 or #12 or #13 or #14 or #15 or #16

#18 animals[mh] NOT human[mh]

#19 #17 NOT #18

#20 #6 and # 9 and #19

**Table B in S1 File. Search strategy for EMBASE**

1. 'dental caries'/exp
2. 'white spot lesion':ab,ti OR 'white spot':ab,ti OR caries:ab,ti OR carious:ab,ti
3. #1 or #2
4. 'deciduous tooth'/exp
5. 'primary teeth':ab,ti OR 'primary dentition*':ab,ti OR 'primary tooth':ab,ti OR 'deciduous tooth':ab,ti OR 'deciduous teeth':ab,ti OR 'milk teeth':ab,ti OR 'milk tooth':ab,ti OR 'baby tooth':ab,ti OR 'baby teeth':ab,ti
6. #4 or #5
7. 'randomized controlled trial'/exp
8. randomized:ab,ti OR randomly:ab,ti OR placebo:ab,ti OR trial:ab,ti
9. #7 or #8
10. #3 and #6 and #9

**Table C in S1 File. Search strategy for Cochrane library**

1. MeSH descriptor: [Tooth Demineralization] explode all trees
2. MeSH descriptor: [Dental Caries] explode all trees
3. MeSH descriptor: [Tooth Remineralization] explode all trees
4. "white spot lesion" or "white spot":ti,ab,kw (Word variations have been searched)
5. caries or carious:ti,ab,kw (Word variations have been searched
6. #1 or #2 or #3 #4 or #5
7. MeSH descriptor: [Tooth, Deciduous] explode all trees
8. "primary teeth" or "primary dentition*" or "primary tooth" or "deciduous tooth" or "deciduous teeth" or "milk teeth" or "milk tooth" or "baby tooth" or "baby teeth":ti,ab,kw (Word variations have been searched
9. #7 or #8
10. "randomized controlled trial" or "controlled clinical trial":pt (Word variations have been searched)
11. randomized or placebo or randomly or trial:ti,ab,kw (Word variations have been searched)
12. MeSH descriptor: [Clinical Trials as Topic] explode all trees
13. #10 or #11 or #12
14. #6 and #9 and #13

**Table D in S1 File. Search strategy for Web of Science**

1. Topic: “tooth remineralization” or “tooth demineralization” or caries OR carious or “white spot lesion” OR “white spot”
2. Topic: “primary teeth” or “primary dentition*” or “primary tooth” or “deciduous tooth” or “deciduous teeth” or “milk teeth” or “milk tooth” or “baby tooth” or “baby teeth”
3. Topic: randomized or placebo or randomly or trial
4. #1 and #2 and #3

**Table E in S1 File. Search strategy for CBM**

[((("龋齿"[加权:不扩展]) AND "牙, 乳"[加权:不扩展]) AND 随机) AND 对照](javascript:toDoRelimitSearch();)

**Table F in S1 File. Search strategy for CNKI**

1. 主题： 龋齿 or 龋 or 脱矿 or 牙面白斑

2. 主题： 乳牙 or 儿童

3. 主题： 随机对照

4. #1 and #2 and #3
