# Supplementary material for: Effect of non-fluoride agents on the prevention of dental caries in primary dentition: A systematic review
Source: PLoS One. 2017 Aug 7;12(8):e0182221. doi: 10.1371/journal.pone.0182221 (PMC5546704; doi:10.1371/journal.pone.0182221)
Supplement: S2 Table — (DOCX) [file pone.0182221.s003.docx]

**S2 Table. Articles excluded from this review**

| **Article** | **Reason for exclusion** |
| --- | --- |
| 1.Cantore R, Petrou I, Lavender S, Santarpia P, Liu Z, Gittins E, et al (2013). In situ clinical effects of new dentifrices containing 1.5% arginine and fluoride on enamel de- and remineralization and plaque metabolism. J Clin Dent 24 Spec no A:A32-44. | *In situ* study |
| 2.Kraivaphan P, Amornchat C, Triratana T, Mateo LR, Ellwood R, Cummins D, et al (2013). Two-year caries clinical study of the efficacy of novel dentifrices containing 1.5% arginine, an insoluble calcium compound and 1,450 ppm fluoride. Caries Res 47(6):582-90. | Compare the anti-caries efficacy on permanent dentition |
| 3.Achong RA, Briskie DM, Hildebrandt GH, Feigal RJ, Loesche WJ(1999). Effect of chlorhexidine varnish mouthguards on the levels of selected oral microorganisms in pediatric patients. Pediatr Dent 21(3): 169-75. | Focus on the effect of chlorhexidine on oral microorganisms |
| 4.Chestnutt IG(2006). Chlorhexidine varnish has caries-reducing potential. Evid Based Dent 7(4):93. | Summary of one included study(Du,2006) |
| 5.Plotzitza B, Kneist S, Berger J, Hetzer G. Efficacy of chlorhexidine varnish applications in the prevention of early childhood caries(2005). Eur J Paediatr Dent 6(3):149-54. | Not RCT |
| 6. James P, Parnell C, Whelton H (2010). The caries-preventive effect of chlorhexidine varnish in children and adolescents: a systematic review. Caries Res 44(4):333-40. | Review article |
| 7.Riley JL 3rd, Gordan VV, Rouisse KM, McClelland J, Gilbert GH (2011). Dental Practice-Based Research Network Collaborative Group. Differences in male and female dentists' practice patterns regarding diagnosis and treatment of dental caries: findings from The Dental Practice-Based Research Network. J Am Dent Assoc 142(4):429-40. | Not RCT |
| 8.Aaltonen AS, Suhonen JT, Tenovuo J, Inkilä-Saari I (2000). Efficacy of a slow-release device containing fluoride, xylitol and sorbitol in preventing infant caries. Acta Odontol Scand 58(6):285-92. | Inclusion criterion for intervention and control not met |
| 9.Isokangas P, Söderling E, Pienihäkkinen K, Alanen P (2000). Occurrence of dental decay in children after maternal consumption of xylitol chewing gum, a follow-up from 0 to 5 years of age. J Dent Res 79(11):1885-9. | Focus on maternal use of xylitol chewing on their children’s caries prevalence |
| 10.Thorild I, Lindau B, Twetman S(2006). Caries in 4-year-old children after maternal chewing of gums containing combinations of xylitol, sorbitol, chlorhexidine and fluoride. Eur Arch Paediatr Dent 7(4):241-5. | Focus on maternal use of chewing gums containing combinations of xylitol, sorbitol, chlorhexidine and fluoride on their children’s caries prevalence |
| 11.Milgrom P, Tut OK(2009). Evaluation of Pacific Islands Early Childhood Caries Prevention Project: Republic of the Marshall Islands. J Public Health Dent 69(3):201-3. | Inclusion criterion for intervention and control not met |
| 12.Milgrom P, Ly KA, Tut OK, Mancl L, Roberts MC, Briand K, Gancio MJ(2009). Xylitol pediatric topical oral syrup to prevent dental caries: a double-blind randomized clinical trial of efficacy. Arch Pediatr Adolesc Med 163 (7):601-7. | Inclusion criterion for intervention and control not met |
| 13.Mickenautsch S, Yengopal V(2012). Effect of xylitol versus sorbitol: a quantitative systematic review of clinical trials. Int Dent J 62(4):175-88. | Review article |
| 14.Taipale T, Pienihäkkinen K, Alanen P, Jokela J, Söderling E(2013). Administration of Bifidobacterium animalis subsp. lactis BB-12 in early childhood: a post-trial effect on caries occurrence at four years of age. Caries Res 47(5):364-72. | Inclusion criterion for control not met |
| 15.Mickenautsch S, Yengopal V (2012). Effect of xylitol versus sorbitol: a quantitative systematic review of clinical trials. Int Dent J 62(4):175-88. | Review article |
| 16.Chi DL, Tut O, Milgrom P (2014). Cluster-randomized xylitol toothpaste trial for early childhood caries prevention. J Dent Child (Chic) 81(1):27-32. | Inclusion criterion for control not met |
| 17.Honkala S, Runnel R, Saag M, Olak J, Nõmmela R, Russak S, et al(2014). Effect of erythritol and xylitol on dental caries prevention in children. Caries Res 48(5):482-90. | Inclusion criterion for intervention and control not met |
| 18.Yang G, Hou X, Guo L, Liu C(2005). Propolis gel on caries preventing.(article in Chinese). J Modern Stomatol 19(1):41-42. | Inclusion criterion for control not met |
| 19.Lin J, Zhao W, Lin J (2015). Fluoride varnish comblined with chlorhexidine gel reducesthe recurrence of severe early childhood caries. (article in Chinese). Chin J Conserv Dent 25 (2):107-109. | Not RCT |
| 20.[Mäkinen KK](http://www.ncbi.nlm.nih.gov/pubmed/?term=M%C3%A4kinen%20KK%5BAuthor%5D&cauthor=true&cauthor_uid=8946097), [Hujoel PP](http://www.ncbi.nlm.nih.gov/pubmed/?term=Hujoel%20PP%5BAuthor%5D&cauthor=true&cauthor_uid=8946097), [Bennett CA](http://www.ncbi.nlm.nih.gov/pubmed/?term=Bennett%20CA%5BAuthor%5D&cauthor=true&cauthor_uid=8946097)(1996). Polyolchewinggums and cariesrates in primarydentition: a 24-monthcohortstudy.[Caries Res](http://www.ncbi.nlm.nih.gov/pubmed/?term=Polyol+chewing+gums+and+caries+rates+in+primary+dentition%3A+a+24-month+cohort+study) 30:408-17. | Not RCT |
| 21.Zhang H, Li Y, Zhang Z(2016). Effects of different caries prevention methods taken by mother on caries incidence of infant. (article in Chinese)J Modern Stomatol 30(1):28-30. | Focus on the effect of maternal use of different preventive methods on dental caries in children |
| 22.Su B, Lu Y, Ding L(2007). Clinical Effect of Cervitec on Caries Prevention in Children. (article in Chinese)Journal of Dental Prevention and Treatment 15(1)1:15-17. | Compare the anti-caries efficacy on permanent dentition |
| 23.Chandak, Bhondey, Bhardwaj(2016). Comparative evaluation of the efficacy of fluoride varnish and casein phosphopeptide - Amorphous calcium phosphate in reducing Streptococcus mutans counts in dental plaque of children: An in vivo study. [J Int Soc Prev Community Dent](https://www.ncbi.nlm.nih.gov/pubmed/?term=Comparative+evaluation+of+the+efficacy+of+fluoride+varnish+and+casein+phosphopeptide+-+Amorphous+calcium+phosphate+in+reducing+Streptococcus+mutans+counts+in+dental+plaque+of+children%3A+An+in+vivo+study) 6(5):423-429. | Focus on the effect of CPP-ACP on oral microorganisms |
| 24.[Bakhshandeh A](https://www.ncbi.nlm.nih.gov/pubmed/?term=Bakhshandeh%20A%5BAuthor%5D&cauthor=true&cauthor_uid=24460688), [Ekstrand K](https://www.ncbi.nlm.nih.gov/pubmed/?term=Ekstrand%20K%5BAuthor%5D&cauthor=true&cauthor_uid=24460688)(2015). Infiltration and sealing versus fluoride treatment of occlusal caries lesions in primary molar teeth. 2-3 years results. [Int J Paediatr Dent](https://www.ncbi.nlm.nih.gov/pubmed/?term=Infiltration+and+sealing+versus+fluoride+treatment+of+occlusal+caries+lesions+in+primary+molar+teeth.+2-3%C2%A0years+results) 25(1):43-50. | Inclusion criterion for intervention and control not met |
| 25.[Duangthip D](https://www.ncbi.nlm.nih.gov/pubmed/?term=Duangthip%20D%5BAuthor%5D&cauthor=true&cauthor_uid=25888484), [Jiang M](https://www.ncbi.nlm.nih.gov/pubmed/?term=Jiang%20M%5BAuthor%5D&cauthor=true&cauthor_uid=25888484), [Chu CH](https://www.ncbi.nlm.nih.gov/pubmed/?term=Chu%20CH%5BAuthor%5D&cauthor=true&cauthor_uid=25888484), [Lo EC](https://www.ncbi.nlm.nih.gov/pubmed/?term=Lo%20EC%5BAuthor%5D&cauthor=true&cauthor_uid=25888484)(2015). Non-surgical treatment of dentin caries in preschool children--systematic review. [BMC Oral Health](https://www.ncbi.nlm.nih.gov/pubmed/?term=Non-surgical+treatment+of+dentin+caries+in+preschool+children+-+systematic+review) Apr 3;15:44. | Review article |
