# Supplementary material for: Effect of non-fluoride agents on the prevention of dental caries in primary dentition: A systematic review
Source: PLoS One. 2017 Aug 7;12(8):e0182221. doi: 10.1371/journal.pone.0182221 (PMC5546704; doi:10.1371/journal.pone.0182221)
Supplement: S3 Table — (DOCX) [file pone.0182221.s004.docx]

**S3 Table. Risk of bias in included studies**

| **Acevedo, A.M /2008** | | | |  |
| --- | --- | --- | --- | --- |
| Item | Authors’ judgment | Description | |  |
| Random sequence generation | Unclear | Comment: insufficient information about the sequence generation process  Quote: “A double-blinded, randomized and controlled clinical trial of one year’s duration was initiated in children… Children entered into the study were randomly divided into two groups (A and B), with distribution being performed on the basis of matching the DMFS (decayed, missing, filled surfaces) levels of their first permanent molars as closely as possible.” | |  |
| Allocation concealment | Unclear | Comment: the method of concealment is not described | |  |
| Blinding of participants and personnel | Unclear | Comment: whether the personnel is blinded or not is unclear.  Quote: “A double-blinded, randomized and controlled clinical trial of one year’s duration was initiated in children.” “Packaging and appearance of both types of mints were identical, except for their A and B designations.” | |  |
| Blinding of outcome assessment | Yes | Quote: “The examiner performing the clinical examinations did not know which group was which, or which of products A and B contained CaviStat. Dental radiographs were not taken.” | |  |
| Incomplete outcome data addressed | Unclear | Comment: insufficient information regarding the number of and reasons for patients’ failure to follow up in each group  Quote: “Out of the 200 children selected, 195 ultimately finished the one-year study with complete data.” | |  |
| Free of selective reporting | No | Comment: the total number of initial lesions is not reported. Adverse events were not reported which should be considered an important outcome in arginine trials.  Quote: “The caries experience was expressed as DMFS, and the total number of initial lesions was calculated only for the first permanent molars by summing the total number of such demineralization lesions detected before and after drying the enamel surface.” | |  |
| Losses to follow-up less than 20% | Yes | Quote: “Out of the 200 children selected, 195 ultimately finished the one-year study with complete data.” | |  |
| Diagnosis reliability | Yes | Quote: “Re-examination of 10% of the subjects was done by the single examiner, and the data were used to determine her consistency by Cohen Kappa analysis.10 Subjects were randomly selected at different times in the study for this re-examination. The Kappa value obtained before the final examination (January, 2006) for the first group of subjects was 0.99, and for the second group (May, 2006) it was 0.99 as well.” | |  |
| Baseline characteristics balance | Yes | Quote: “These numbers were essentially the same, because the matching of the two groups of subjects at baseline was performed to try to distribute subjects evenly.” | |  |
| Free of contamination | Unclear | Comment: no strategies were mentioned to avoid contamination between groups | |  |
| **Gisselsson, H/1994** | | | |  |
| Item | Authors’ judgment | Description | |  |
| Random sequence generation | Unclear | Comment: insufficient information about the sequence generation process  Quote:“the 117 children participating in the professional flossing-gel program were randomly distributed in two groups, here called the chlorhexidine(gel) group and the placebo(gel) group.” | |  |
| Allocation concealment | Yes | Quote:“in the chlorhexidine group(n=59),a 1% chlorhexidine gel(Hibitane Dental, ICI, Macclesfield, UK) was used and in the placebo group(n=58)an identical gel without chlorhexidine, produced by the same manufacture. The gels were marked A and B and the code was not broken until the caries data had been analyzed.” | |  |
| Blinding of participants and personnel | Yes | Comment: insufficient information to make a judgment about the blinding of participants and personnel.  Quote:“in the chlorhexidine group(n=59),a 1% chlorhexidine gel(Hibitane Dental, ICI, Macclesfield, UK) was used and in the placebo group(n=58)an identical gel without chlorhexidine, produced by the same manufacture. The gels were marked A and B and the code was not broken until the caries data had been analyzed.” | |  |
| Blinding of outcome assessment | Unclear | Comment: insufficient information to make a judgement.  Quote:“At baseline, when the children were 4 years old, only clinical examination were performed …in the two gel group, it was carried out by one and the same dentist(H.G.) and in the control group, by 3 other dentists at the same clinic.”“All registration in the two experimental groups as well as in the control group were carried out by one of the author (H.G), without knowing to which group the children belong to. Enamel and dentin caries were registered separately.” | |  |
| Incomplete outcome data addressed | Unclear | Comment: insufficient information regarding the number of and reasons for patients’ failure to follow up in the test and placebo group  Quote: (for test and placebo group): “The total population of children born in 1983…Four could not participate because of treatment difficulties, 3 were about to move, and the parents of 3 children were not interested…Thus, 117 children out of 132 (89%) fulfilled the 3-year study.” (for control group): “Because of drop-outs, mainly for the same reasons as described above, only 116 of 131(89%) remained at the final examination at 7 years of age.” | |  |
| Free of selective reporting | unclear | Comment: Mean number of decayed, extracted and filled teeth and approximal tooth surfaces at baseline and final examination were reported. However, adverse events were not reported which should be considered an important outcome in chlorhexidine trials. | |  |
| Losses to follow-up less than 20% | Yes | Quote :(for test and placebo group):“Thus,117children out of the 132 (89%) fulfilled the 3-year study.”(for control group): “Because of drop-outs, mainly for the same reasons as described above, only 116 of 131(89%) remained at the final examination at 7 years of age.” | |  |
| Diagnosis reliability | Yes | Quote: “In order to evaluate the reliability of diagnose based on bitewing radiographs, 10% of the X-films from the final exanimation…for a second exanimation.” “Differences in the number of lesions in the enamel were recorded in 6 children and 1 child was found to have one lesion less…None of the caries-free children were diagnosed to have caries at the second assessment.” | |  |
| Baseline characteristics balance | Yes | Quote:“the distribution of children with dentin caries and fillings on the approximal tooth surfaces at baseline,…No statistically significant differences were found between the groups.” | |  |
| Free of contamination | Yes | Comment: the test and control gel were applied by dentists | |  |
| **Tai ,B.J /2003** | | | |  |
| Item | Authors’ judgment | Description | |  |
| Random sequence generation | Unclear | Comment: insufficient information about the sequence generation process  Quote：“随机抽取武汉市3所幼儿园和1所小学的1250名学生（3-7岁）参加本研究。按照年龄分为 3岁年龄组（ 327人）、 5岁年龄组（517人）和 6-7岁年龄组（487 人）。每个年龄组按照班级又随机分为实验组（洗必泰涂料组）、阴性对照组（山达胶涂料组）和空白对照组（安慰剂组），各组间性别差异无显著意义。” | |  |
| Allocation concealment | Unclear | Comment: the method of concealment is not described | |  |
| Blinding of participants and personnel | Unclear | Comments: whether participants were really blinded to group assignment was doubtful due to special taste of the test vanish.  Quote: “本实验采用双盲法，即检查者和受试者均不知道分组情况。”“92%学生认为所涂药物的味道较差，但只有13%的学生认为涂药过程非常难受。 86%的学生表示愿意继续接受该项预防措施。”“几乎大部分学生认为洗必泰涂料味道较差。” | |  |
| Blinding of outcome assessment | Yes | Comment: double blind  Quote: “本实验采用双盲法，即检查者和受试者均不知道分组情况。” | |  |
| Incomplete outcome data addressed | Yes | Quote: “按照年龄分为 3岁年龄组（ 327人）、 5岁年龄组（517人）和 6-7岁年龄组（487 人）。2年后由于样本流失、或者未能参加每次涂药…各年龄组样本失访率分别为12.8% ,13.0%和3.2% 。” | |  |
| Free of selective reporting | Yes | Comment: mean dmfs scores of three groups at baseline and after 24 months were reported | |  |
| Losses to follow-up less than 20% | Yes | Quote: “各年龄组样本失访率分别为12.8% 、 13.0%和3.2% 。” | |  |
| Diagnosis reliability | unclear | Comment: the inter-examiner reliability were not reported.  Quote: “研究前对4名检查者进行培训，统一标准，经标准一致性检验,4名医生的Kappa 值分别为1.0 、0.97 、0.93 和0.91.” | |  |
| Baseline characteristics balance | Yes | Quote: “实验前各组间龋均、患龋率、龋面均均无统计学差异.” | |  |
| Free of contamination | Yes | Comment: the test vanish and placebo varnish were applied by dentists rather than by children or their parents. | |  |
| **Baca, P /2004** | | | |  |
| Item | Authors’ judgment | Description |  |  |
| Random sequence generation | Unclear | Comment: insufficient information about the sequence generation process  Quote: “Five elementary schools were randomly selected from all 21 elementary schools in the northern section of the city.” “The classes were randomly designated as control group or varnish group.” |  |  |
| Allocation concealment | Unclear | Comment: the method of concealment is not described |  |  |
| Blinding of participants and personnel | No | Quote: “The children were informed that they should not drink or eat for 3 hours (for this reason, application took place after their recess), brush their teeth until the following day, or use dental floss for 1 week. The control group received no treatment other than clinical examination.” “The examining clinician was not blinded to the group that the participants belonged to, which may be a study limitation.” |  |  |
| Blinding of outcome assessment | No | Quote: “All examinations, which were not performed in a blinded fashion, were carried out by the same dentist.” |  |  |
| Incomplete outcome data addressed | Unclear | Comment: insufficient information regarding the number of and reasons for patients’ failure to follow up in each group  Quote: “Out of the 229 schoolchildren with written consent to participate in the study, 48 (21%) were lost to the follow-up, usually because they changed to a different school or their parents decided to interrupt the program. A total of 181 children participated for the full 24 months: 86 in the chlorhexidine varnish group and 95 in the control group. ” |  |  |
| Free of selective reporting | Yes | Comment: caries lesion prevalence and incidence at baseline and after 24 months were reported. |  |  |
| Losses to follow-up less than 20% | No | Quote: “Out of the 229 schoolchildren with written consent to participate in the study, 48 (21%) were lost to the follow-up, usually because they changed to a different school or their parents decided to interrupt the program. A total of 181 children participated for the full 24 months: 86 in the chlorhexidine varnish group and 95 in the control group.” |  |  |
| Diagnosis reliability | Yes | Quote: “The kappa coefficient for intra-observer and inter-observer reliability was >0.63, which is considered satisfactory on the scale of Landis and Koch.” |  |  |
| Baseline characteristics balance | Yes | Quote: “There were no significant differences at baseline in gender, age,socioeconomic level, number of teeth present, or caries lesion index between those completing the study and the lost cases (results not shown).” “At baseline, there were no significant differences between these 2 groups in age, number of teeth present, or caries lesion index (Table 1).”“ At baseline, there were no significant differences between the groups in the caries lesion indexes for primary molars (dftm and dfsm)” |  |  |
| Free of contamination | Yes | Quote: “The applications were carried out in the schools by 2 dentists using portable equipment…. The control group received no treatment other than clinical examination.” |  |  |
| **Du, M.Q/2006** | | | |  |
| Item | Authors’ judgment | Description | | |
| Random sequence generation | Yes | Quote:“Four kindergartens in the study district were chosen randomly in 1999, and all children aged 4-5 yrs in these kindergartens were invited to participate in the study.”“We randomly divided the children into two groups, based on the class they attended, by drawing a card from a bag.” | | |
| Allocation concealment | Unclear | Comment: the method of concealment is not described | | |
| Blinding of participants and personnel | Yes | Comments: whether participants were really blinded to group assignment was doubtful due to special taste of the test vanish.  Quote: “This double-blinded, randomized, placebo-controlled clinical trial was carried out according to the guidelines for good clinical practice”. “The children, their parents, and the kindergarten staff were blinded as to the group assignment of the children.” “The 2 varnishes were put into bottles that had the same appearance, to ensure blindness of the trial…the chlorhexidine or the placebo varnish was applied to all surfaces of the primary molars of the children, according to their group assignment, by two dentists who did not know the content of the varnish.” “After 2 yrs, 44 children (13%) were lost to follow-up, because some (n= 31) had moved to other kindergartens, and some (n = 13) objected to the taste of the varnish and refused to be examined.” | | |
| Blinding of outcome assessment | Yes | Quote: “Two calibrated dentists who did not know the group assignment of the children performed all clinical examinations.” | | |
| Incomplete outcome data addressed | Unclear | Comment: Reasons given for losses to follow-up, but not clear in which groups  Quote: “After 2 yrs, 44 children (13%) were lost to follow-up, because some (n= 31) had moved to other kindergartens, and some (n = 13) objected to the taste of the varnish and refused to be examined. The baseline mean dmfs-molar scores of the children who dropped out were similar to those of the children who remained in the study (2.6 *vs.* 2.7; *t* test, p > 0.05). The result presented below was based on the 290 children who remained in the study after 2 yrs (155 in the test group and 135 in the placebo group).” | | |
| Free of selective reporting | Yes | Comment: mean dmfs-molar scores of two groups at baseline and after 24 months were reported | | |
| Losses to follow-up less than 20% | Yes | Quote: “After 2yrs, 44 children (13%) were lost to follow-up, because some (n= 31) had moved to other kindergartens, and some (n = 13) objected to the taste of the varnish and refused to be examined.” | | |
| Diagnosis reliability | Yes | Comment: results of the duplicate examinations showed that the inter-examiner reliability in assessing the tooth surface status was very good (Kappa > 0.90). | | |
| Baseline characteristics balance | Yes | Comment: The respective baseline mean dmfs molar scores of these two groups of children were 2.8 and 2.6(Table), and the difference was not statistically significant (*t* test, p > 0.05). | | |
| Free of contamination | Yes | Comment: the test vanish and placebo varnish were applied by dentists rather than by children  Quote: “Seven classes of children were assigned to the chlorhexidine varnish group (test), and seven classes were assigned to the placebo control group…the chlorhexidine or the placebo varnish was applied to all surfaces of the primary molars of the children in the kindergartens, according to their group assignment, by two dentists who did not know the content of the varnish.” | | |
| **Amorim, R. G/2008** | | | |  |
| Item | Authors’  judgment | Description | | |
| Random sequence generation | Unclear | Comment: insufficient information about the sequence generation process  Quote: “A total of 80 caries-active preschool children (3–5 years) were randomly divided into four groups.”“After that, the 80 children were randomly divided into four groups.” | | |
| Allocation concealment | Unclear | Comment: the method of concealment is not described | | |
| Blinding of participants and personnel | No | Quote: “Control group. No treatment was performed, except restorative treatment when necessary…The test groups received the recommendation not to ingest food or drinks for 1 hour after varnish applications and not to brush their teeth on the day of application to prevent chemical interactions among the dentifrice components and the substances in the varnish formulations…In G4, the returns (T2 and T3) were scheduled from the last restorative treatment session.” | | |
| Blinding of outcome assessment | Unclear | Comment: no detailed information was found regarding the blinding of outcome assessment | | |
| Incomplete outcome data addressed | Unclear | Comment: insufficient information regarding the reasons for patients’ failure to follow up in each group.  Quote: “Sample loss was around 5%, because of the 80 children in the initial sample, 78 were examined at time T2, and 76 at time T3.” | | |
| Free of selective reporting | Unclear | Comment: the mean variation of visible plaque index and white spot lesion with time intervals were all reported. However, adverse events were not reported which should be considered an important outcome in chlorhexidine trials. | | |
| Losses to follow-up less than 20% | Yes | Quote: “Sample loss was around 5%, because of the 80 children in the initial sample, 78 were examined at time T2, and 76 at time T3.” | | |
| Diagnosis reliability | Yes | Quote: “The degree of intra-examiner agreement (kappa) was 0.964 (T1).” | | |
| Baseline characteristics balance | Yes | Quote: “The four groups presented very similar mean VPI and WS values (not statistically significant) at the initial clinical exam (T1), as shown in Table 1.” | | |
| Free of contamination | Yes | Comment: the test vanish and placebo varnish were applied by dentists rather than by children or their parents. | | |
| **Plonka, K. A./2013** | | | |  |
| Item | Authors’ judgment | Description | | |
| Random sequence generation | Yes | Quote: “CPP-ACP topical cream (Tooth Mousse, GC, Tokyo Japan), 0.12% CHX gel (Curasept, Curaden Swiss, Saranno, Switzerland), or no product (allocation ratio=1:1:1), blindly selected by each mother randomly picking a color-coded stick from an opaque bag.” | | |
| Allocation concealment | Unclear | Comment: the method of concealment is not described | | |
| Blinding of participants and personnel | No | Comment: the blinding of personnel is almost impossible because interventions were different.  Quote:“At recruitment, participants were randomized to use either 10% CPP-ACP topical cream (Tooth Mousse, GC, Tokyo,Japan), 0.12% CHX gel (Curasept, Curaden Swiss, Saranno, Switzerland), or no product (allocation ratio=1:1:1), blindly selected by each mother randomly picking a color-coded stick from an opaque bag.” | | |
| Blinding of outcome assessment | Yes | Quote: “All children were examined at their homes at 6, 12, and 18 months of age, and at the community dental clinic at 24 months by examiners who were blinded to treatment group” | | |
| Incomplete outcome data addressed | Yes | Comment: missing outcome data balanced in numbers across intervention groups, with similar reasons for missing data across groups  Quote: “At 24-months-old, the retention rate was 87% (542/622), with the main reason for dropout being inability to contact and with consistent drop out numbers amongst all groups.” | | |
| Free of selective reporting | Yes | Comment: the prevalence of MS and LB in the CPP/ACP,CHX, and SC groups by age of 6,12 and 18 month were reported. | | |
| Losses to follow-up less than 20% | Yes | Quote: “At 24-months-old, the retention rate was 87% (542/622), with the main reason for dropout being inability to contact and with consistent drop out numbers amongst all groups” | | |
| Diagnosis reliability | Yes | Quote: “The mean intra- and inter-examiner kappa statistics were 0.94 and 0.86, respectively.” | | |
| Baseline characteristics balance | Yes | Quote: “The groups were tested for comparability for a range of characteristics, such as: mother’s age at the child’s birth… There were no statistically significant differences among the groups in all these characteristics at all contact times.” | | |
| Free of contamination | Yes | Quote: “Mothers were provided with toothpastes, toothbrushes, and test products free of charge at the 6-month visit and instructed to use them as soon as the teeth emerge. All children were examined at their homes at 6, 12, and 18 months of age, and at the community dental clinic at 24 months by  examiners who were blinded to treatment group.” | | |
| **Pukallus, M. L/2013** | | | |  |
| Item | Authors’ judgment | Description | | |
| Random sequence generation | Yes | Quote: “Randomisation into the CHX or control groups was performed at recruitment by every mother selecting a colour-coded stick out of an opaque bag (allocation ratio 1 : 1).” | | |
| Allocation concealment | Unclear | Comment: the method of concealment is not described. | | |
| Blinding of participants and personnel | No | Comment: the blinding of personnel and participants is almost impossible because intervention and control method were different.  Quote: “Children were randomized to receive either (i) once daily application of 0.12% CHX gel (Curasept_; Curaden Swiss, Saranno, Switzerland) as soon as the first teeth erupted, or (ii) no product (study control). The mothers of both CHX and study control groups were instructed to brush twice daily with a smear of low-dose fluoride dentifrice (304% fluoride) on a child’s toothbrush (My First Toothpaste_; Colgate Oral Care, Sydney, NSW, Australia) as soon as the first teeth emerged.” | | |
| Blinding of outcome assessment | Yes | Quote: “The examinations were conducted in the community dental clinic by seven calibrated examiners who were blinded to the treatment received.” | | |
| Incomplete outcome data addressed | No | Comment: missing outcome data is not balanced in numbers across intervention groups, and the plausible effect size among missing outcomes may have a clinically relevant impact on observed effect size.  Quote: “At the examination visit at 24 months, there were no statistically significant differences in retention rates for the CHX (55%) and study controls (65%). The main reasons for drop-out from both groups were inability to contact the mother, and her relocation beyond a reasonable distance from the research centre (Table 2).” | | |
| Free of selective reporting | Yes | Comment: caries rate after 24 months in the CHX and control groups were reported | | |
| Losses to follow-up less than 20% | No | Quote: “At the examination visit at 24 months, there were no statistically significant differences in retention rates for the CHX (55%) and study controls (65%).” | | |
| Diagnosis reliability | Yes | Quote: “The mean kappa statistics from the intra- and inter-examiner variability tests for the examination of carious lesions were 0.91 and 0.86 which indicated high consistency.” | | |
| Baseline characteristics balance | Yes | Quote: “The randomisation of the groups resulted in comparable groups with regard to sociodemographic and caries-risk variables (Table 1).” | | |
| Free of contamination | Yes | Comment: the daily application of CHX was conducted at home by mother, rather than by children themselves. | | |
| **Sitthisettapong, T /2012** | | | |  |
| Item | Authors’ judgment | Description | | |
| Random sequence generation | No | Comment: the method of random sequence generation is inadequate  Quote: “After baseline ICDAS, children were systematically allocated to two groups, and the allocation of treatment to each group was done randomly.”“ Randomly, those with odd ID numbers were assigned to the experimental treatment, and those with even ID numbers to the control treatment.” | | |
| Allocation concealment | Yes | Quote: “The allocation of experimental or control group was determined by an assistant who was not involved in the clinical aspects of the study, to reduce potential biases” “The paste code was retained by the manufacturer and broken only after analysis of the study results” | | |
| Blinding of participants and personnel | Yes | Quote: “those in the control group received a placebo paste that had identical packaging, color, and taste, since it was prepared and provided by the same manufacturer. All staff, teachers and participating children were blinded to the group assignment and did not know which paste is more effective in caries prevention. The paste code was retained by the manufacturer and broken only after analysis of the study results. ” | | |
| Blinding of outcome assessment | Yes | Quote: “All children were examined at the nursery schools by one pediatric dentist who was blinded to the child’s group assignment.” | | |
| Incomplete outcome data addressed | Yes | Comment: missing outcome data balanced in numbers across intervention groups, with similar reasons for missing data across groups  Quote: “150 were assigned to the experimental condition and 146 to the control condition. …the drop-out rates of the experimental and the control groups were 22.0% and 23.3% (p = 0.79). Two major reasons for drop-out were children’s transfer to higher level schools and families moving out of the area (Fig.)””Comparison of background characteristics and baseline dmfs/dmft between the remaining 213 children and the 86 children who dropped out demonstrated no statistically significant difference, except for gender, with significantly more girls in the drop-out group (p = 0.036).” | | |
| Free of selective reporting | Yes | Comment: mean number of caries outcome measures at baseline, 6 mos, and 1 yr are all reported | | |
| Losses to follow-up less than 20% | No | Quote: “After 1 yr, 229 children remained in the study; the drop-out rates of the experimental and the control groups were 22.0% and 23.3% (p = 0.79). Two major reasons for drop-out were children’s transfer to higher level schools and families moving out of the area (Fig.).” | | |
| Diagnosis reliability | Yes | Quote: “Intra-examiner reliability, based on approximately 10% of the children and computed by the weighted kappa statistic at baseline, 6 mos, and 1 yr, was high: 0.80, 0.79, and 0.87, respectively.” | | |
| Baseline characteristics balance | Yes | Quote: “At baseline, the two groups were comparable in terms of potential confounders with respect to age, gender,eating behaviors, toothbrushing behavior, and parents’ socio-economic status…The baseline dmfs/dmft score for both groups was high, 9.1/4.3 and 9.4/4.3, respectively…The difference in baseline dmfs/dmft observed between two groups was not statistically significant (p = 0.88 and p = 0.91) (Table 1).” | | |
| Free of contamination | Yes | Comment: the test paste and placebo paste were applied by teacher rather than by children.  Quote: “The paste was applied by trained teachers every school day, using each child’s coded paste, following toothbrushing with fluoridated toothpaste after lunch.” | | |
| **Memarpour, M/2015** | | | |  |
| Item | Authors’ judgment | Description | | |
| Random sequence generation | Yes | Quote: “The 140 children were randomized into 4 groups with 35 in each group…At baseline and follow-up appointments, a staff member at the health clinic selected children by means of a block randomization method with the help of a random number table.” | | |
| Allocation concealment | Unclear | Comment: the method of concealment is not described | | |
| Blinding of participants and personnel | No | Comment: the intervention method between groups were different, so it is almost impossible to blind the personnel.  Quote: “Group 1(control) received no preventive intervention. In group 2,there was oral hygiene and dietary counseling. In group 3, there was oral hygiene and the application of fluoride varnish at 4, 8 and 12 months after baseline. In group 4, there was oral hygiene and tooth mousse was applied by the parents twice a day over a 12-month period.” | | |
| Blinding of outcome assessment | Yes | Quote: “The examiners and the parents were blinded as to which group each child had been randomly assigned.” | | |
| Incomplete outcome data addressed | Yes | Comment: missing outcome data balanced in numbers across intervention groups, with similar reasons (transfer to another school) for missing data across groups  Quote：“Follow-up appointments were scheduled for each child at 4, 8 and 12 months after the baseline examination. During follow-up, 18 children were excluded from the study (fig. 1 ).” | | |
| Free of selective reporting | Yes | Comment: changes in mean WSL size and the dmft index in all groups during follow-up were reported | | |
| Losses to follow-up less than 20% | Yes | Quote:“In this 1-year,randomized clinical trial, 140 children aged 12–36 months with WSL in the anterior maxillary teeth were selected and randomly divided into 4 groups of 35 children each.” “During follow-up, 18 children were excluded from the study (fig. 1 ).” | | |
| Diagnosis reliability | Yes | Quote: “A high level of intra-examiner reliability was achieved for the identification and measurement of WSL according to a weighted kappa statistic of 85% and an intra-class correlation of r = 0.88 in 20 randomly selected children.” | | |
| Baseline characteristics balance | Unclear | Comment: no detailed information was found regarding the baseline caries level of the subjects  Quote：“At baseline, 140 children (mean age: 21.20 ± 6.76 months) with a total of 483 active WSL were enrolled…There were no significant differences between groups in terms of sex ratio (p = 0.280) or mean age (p = 0.657).” | | |
| Free of  contamination | Yes | Comment: the preventive method were applied by dentists.  Quote: “Two dentists (E.F. and S.D.) performed all of the dental examinations at the baseline and follow-up appointments.” “At each follow-up appointment, one of the examiners first measured WSL and then recorded the dmft index. One of the preventive methods was applied by the other examiner (detailed below).” | | |
| **Cao,H.Z/2007** |  |  | | |
| Item | Authors’ judgment | Description | | |
| Random sequence generation | Unclear | Comment: insufficient information about the sequence generation process  Quote：“选择上海市2～5 岁儿童606 例, 分布于4 所幼儿园, 随机抽取.其中2 所为试验组, 另2 所为对照组” | | |
| Allocation concealment | Unclear | Comment: the method of concealment is not described | | |
| Blinding of participants and personnel | No | Comments: participants and personnel were not blinded.  Quote: “试验组: 嘱儿童用清水漱口, 检查者在棉卷隔湿下, 用浸没涂膜的小棉球全口涂布至各乳牙面, 30s时间内涂膜在牙面成膜, 嘱涂膜后30min 内不漱口和进食。每半年的第1 个月开始涂布, 1 年中共涂布2 次。对照组: 对照组儿童不作任何处理。” | | |
| Blinding of outcome assessment | Yes | Comment: Single blind  Quote: “采用双盲法进行检查和统计,即检查记录者和数据统计处理者均不知试验分组情况。” | | |
| Incomplete outcome data addressed | Unclear | Comments: missing outcome data not balanced in number and the reasons for patients’ failure to follow up in each group is unclear.  Quote: “试验初始时, 试验组和对照组儿童人数共606名, 其中试验组328 名、对照组278 名。1 年后因转学等原因, 复查时试验组296 名, 对照组265 名。” | | |
| Free of selective reporting | Unclear | Comment: mean dmfs scores of two groups at baseline and after one year were reported. However,adverse events were not reported which should be considered an important outcome in triclosan trials. | | |
| Losses to follow-up less than 20% | Yes | Quote: “试验初始时, 试验组和对照组儿童人数共606名, 其中试验组328 名、对照组278 名。1 年后因转学等原因, 复查时试验组296 名, 对照组265 名。” | | |
| Diagnosis reliability | Unclear | Comment: the intra-examiner reliability were not reported.  Quote: “2 名检查者均为口腔科专业医师, 具有丰富的临床经验, 经标准一致性试验检验合  格, Kappa 值为0.84。” | | |
| Baseline characteristics balance | Yes | Quote: “试验初始时, 试验组和对照组2 组儿童的患龋率分别为69%和63%, 龋均分别为0.98±2.11 和0.93±2.06, 龋面均分别为1.24±2.60 和1.18±2.28,均无显著差异(P>0.05).” | | |
| Free of contamination | Yes | Comment: the test vanish and placebo varnish were applied by dentists rather than by children or their parents. | | |
| **Oscarson, P/2006** | | | |  |
| item | Authors’ judgment | Description | | |
| Random sequence generation | Unclear | Comment: insufficient information about the sequence generation process.  Quote: “The study had a randomised single-blind prospective design and approved by the local ethical committee of Ume University. After inclusion, the children were randomly assigned to a test or a control group with the exception of twins and siblings that were allocated to the same group.” | | |
| Allocation concealment | Unclear | Comment: the method of concealment is not described | | |
| Blinding of participants and personnel | No | Comment: the participants and personnel were not blinded.  Quote: “The study had a randomised single-blind prospective design…”“The children in the test group were provided with sucking tablets containing 0.48 gram xylitol…The children in the control group were not given any tablets at any time.” | | |
| Blinding of outcome assessment | Yes | Quote: “Caries was registered by tactile and visual examination in a dental chair by two blinded calibrated examiners …” | | |
| Incomplete outcome data addressed | No | Comment: missing outcome data is not balanced in numbers across intervention groups, and the plausible effect size among missing outcomes may have a clinically relevant impact on observed effect size.  Quote: “The attrition was higher in the test group in which 11children abandoned the tablet program or were lost during follow-up due to no-show or relocation. The corresponding number in the control group was 3 children. The mothers of another 3 children in the control group did not turn up for bacteriological samplings.” | | |
| Free of selective reporting | unclear | Comment: Mutans streptococci colonisation and Caries prevalence at baseline and the age of 4 years were reported. However, adverse events were not reported which should be considered an important outcome in xylitol trials. | | |
| Losses to follow-up less than 20% | Yes | Quote: “The total dropout rate during the 2-year study was 10.6%.” | | |
| Diagnosis reliability | Unclear | Comment: the study did not mention the inter/ intra-examiner reliability  Quote：“Caries was registered by tactile and visual examination in a dental chair by two blinded calibrated examiners according to the guidelines of the Public Dental Service in accordance to the WHO criteria [1987]) modified for the primary dentition.” | | |
| Baseline characteristics balance | Yes | Comment: there were no statistically significant differences between test and control groups for any of the baseline measurements.（For details，see Table 1） | | |
| Free of contamination | Yes | Quote:“The material consisted of 132 healthy 2-year-old children, 71 boys and 61 girls and they were assigned to a xylitol tablet (test) group or a non-intervention control group.”“The children in the test group were provided with sucking tablets containing 0.48 gram xylitol …and the parents was asked to give the children one tablet per day at bedtime after tooth-brushing... The children in the control group were not given any tablets at any time.” | | |
| **Zhan, L/2012** | | | |  |
| item | Authors’ judgment | Description | | |
| Random sequence generation | Yes | Quote: “The participants were then randomized to either xylitol-wipe (Spiffies Baby Tooth Wipes^TM^, DR Products Inc., Tucson, AZ, USA) or placebo-wipe groups based on their order of recruitment, using a pre-set computer-generated random number table based on their order of recruitment by ML after a qualification examination.” | | |
| Allocation concealment | Yes | Quote: “The groups were blinded as Groups A and B.”… “Only one investigator (JDBF), who was not involved in any patient contact, dental examinations, and microbiological assays, knew the group assignment.” | | |
| Blinding of participants and personnel | Yes | Quote: “In a double-blinded randomized controlled clinical trial, 44 mothers with active caries and their 6- to 35-month-old children were randomized to xylitol-wipe or placebo-wipe groups”“All the other investigators involved in participant contact, microbiological assays, and statistical analysis were blinded.”“ The placebo wipes were custom-synthesized by Dr. Products Inc. for the study and were identical in appearance and composition, except that there was no xylitol in the placebo wipe. Both wipes were grape-flavored, though the xylitol wipe had a sweeter taste than the placebo wipe.” | | |
| Blinding of outcome assessment | Yes | Quote: “All the other investigators involved in participant contact, microbiological assays, and statistical analysis were blinded.” | | |
| Incomplete outcome data | Yes | Comments: we consider that the attrition bias was low.  Quote: “In the placebo-wipe group, seven mothers reported rejection of wipe-use by the children, resulting in two drop-outs. Four other children dropped from the study because of moving, loss of interest, or family difficulty. In the xylitol-wipe group, two participants dropped from the study due to moving or loss of interest. Two other participants reported non-compliance due to rejection of wipe-use by the child or family difficulty.” “The primary statistical analyses include ITT modeling procedures for new caries lesions… To assess whether the result is sensitive to different assumptions  about the nature of the missing data, we conducted a secondary analysis including caries data on only participants who completed the study.” | | |
| Free of selective reporting | Yes | Comment: appropriate outcome measures were considered and reported. | | |
| Losses to follow-up less than 20% | Yes | Quote: “Forty-four mother-child pairs were recruited for the study (n = 22 for each group. At 1 yr, 20 individuals from the xylitol-wipe group and 17 from the placebo-wipe group completed the study ([Fig. 1](https://www.ncbi.nlm.nih.gov/pmc/articles/PMC3383105/figure/fig1-0022034511434354/)).” | | |
| Diagnosis reliability | Yes | Quote: “Cross-calibration on inter-examiner reliability was performed on seven children (15% of the study population). The two examiners showed 100% agreement on caries scoring, with Kappa = 1 (P < 0.01).” | | |
| Baseline characteristics balance | Yes | Quote: “No statistically significant differences were found in age, caries status, and levels of MS and LB for the mothers and their children between the two groups (P >0.05).）”（Table 1） | | |
| Free of contamination | Yes | Comment: the preventive method were applied by mothers.  Quote: “The wipes were provided to the mothers every 3 mos. Mothers were instructed to use 2 wipes to clean the teeth and gums of the children 3 times daily in addition to daily tooth-brushing.” | | |
| **Lee, W/2015** | | | |  |
| item | Authors’ judgment | Description | | |
| Random sequence generation | Yes | Quote: “The study was designed as a two-group placebo-controlled double-blind cluster (classroom)-randomized clinical trial, with an intervention of 9 months and a total follow-up of 30 months…Each of the five participating schools had half of the classrooms randomized to either xylitol or placebo gummy bear using a computer-generated scheme.” | | |
| Allocation concealment | Unclear | Comment: the method of concealment is not described. | | |
| Blinding of participants and personnel | Yes | Quote: “All children and their parents, investigators, study staff delivering the gummy bear intervention, dental examiners and statisticians were blinded to the treatment assignment”“All gummy bears were formulated to be similar in size, consistency, color and sweetness” | | |
| Blinding of outcome assessment | Yes | Quote: “The purpose of this double-blind, cluster-randomized clinical trial was to examine the effects of xylitol gummy bear snacks on dental caries progression in primary and permanent teeth of inner-city school children… All children and their parents, investigators, study staff delivering the gummy bear intervention, dental examiners and statisticians were blinded to the treatment assignment.” | | |
| Incomplete outcome data | Yes | Comment: missing outcome data balanced in numbers across intervention groups, with similar reasons (given in Figure 1) for missing data across groups  Quote: “A total of 260 children comprised the xylitol group and 265 children the placebo group (37 children did not have baseline exams).” | | |
| Free of selective reporting | Yes | Comment: mean new dmfs/DMFS of different groups at different time points were reported. | | |
| Losses to follow-up less than 20% | No | Quote: “Although the retention rate was approximate 85% at the end of kindergarten(476/562), 67% at the start of 1st grade (378/562), 61% at the end of 1st grade (342/562) and 52% at the exit exam in 2nd grade (295/562), some children were sick at school or absent from school on dental exam dates.” | | |
| Diagnosis reliability | Yes | Quote: “The inter- and intra-rater reliability for the examiners was good to excellent with a kappa >0.70” | | |
| Baseline characteristics balance | Yes | Comments: although participants of xylitol group had significantly higher d3–6mfs compared with the placebo group at baseline，this difference is unlikely to have a distinct impact on outcome assessed.  Quote: “The baseline characteristics (table 1) show that the two groups were similar in gender, ethnicity and cohort. The children assigned to the xylitol group had significantly higher d3–6mfs compared with the placebo group.” | | |
| Free of contamination | Yes | Quote: “Randomization was done at the classroom level to prevent mixing of the treatments (children getting the wrong type of gummy bear) and to simplify the logistics of gummy bear distribution.”“Xylitol or placebo gummy bears were given only within the supervised school environment and were not available to the children when not in school, e.g. during school breaks. Outreach workers in each school dispensed the gummy bears to children and kept a daily gummy bear log to document compliance.” | | |
